# Random amplified microsatellites (RAMS) analysis ascertains genetic variation of *Alternaria alternata* causing black spot disease on *Carya illinoinensis* in South Africa

**DOI:** 10.3389/fgene.2023.1213102

**Published:** 2023-09-27

**Authors:** Conrad Chibunna Achilonu, Marieka Gryzenhout, Gert Johannes Marais, M. Thabang Madisha, Soumya Ghosh

**Affiliations:** ^1^ Department of Plant Sciences, Division of Plant Pathology, Faculty of Natural and Agricultural Sciences, University of the Free State, Bloemfontein, South Africa; ^2^ Department of Genetics, Faculty of Natural and Agricultural Sciences, University of the Free State, Bloemfontein, South Africa; ^3^ Agricultural Research Council (ARC), Pretoria, South Africa

**Keywords:** *Alternaria alternata*, ascomycota fungi, *Carya illinoinensis*, genetic diversity, microsatellites

## Abstract

Limited information regarding the occurrence of black spot disease of pecan (*Carya illinoinensis*), caused by *A. alternata*, in South Africa is known. The pecan industry is growing rapidly, so it is essential to understand the impact of the fungal pathogen to pecan health. In this study, the genetic variation of 364 *A. alternata* isolates was investigated by two RAMS primers (CCA_5_ and CGA_5_). In total, 6,525 alleles were produced, with a minimum of 3,182 alleles on the CGA_5_ primer and maximum of 3,343 alleles for CCA_5_ primer. Further analysis of the primers showed relatively low genetic diversity of *A. alternata* isolate populations, with mean values; (*H* = 0.12) and Shannon’s information index (*I* = 0.20). The analysis of molecular variance (AMOVA) revealed significant differences between populations, with 88% of the genetic variation was found within populations (*Nm* = 3.59, PhiPT = 0.12), and were not significantly different (*p* > 0.001). While 12% variation was observed among populations (*Nm* = 2.89, PhiPT = 0.08) and the estimates were statistically significant (*p* < 0.001). STRUCTURE HARVESTER output showed that *K* value is *K* = 8, where Δ*K* cannot find the true number of populations because of less variation. The dendrogram cluster tree generated by Ward’s analysis unveiled two main distinct clades and 10 sub-clades, revealing similar findings as those of PCoA analysis clusters. Therefore, it was evident that these analyses depicted no distinct relationship between the *A. alternata* isolates and their geographic locations or the prevalence of distribution among the populations.

## Introduction

*Carya illinoinensis* (pecan) is one of the premier tree nut crops experiencing a rapid upsurge in nut production and sales across the global tree nut market (Persistence Market Research, 2018). The revenue growth of the global market is mainly driven by the awareness of various health benefits associated with pecans (Healthline, 2020). The United States of America produces about 52.0% of pecan nuts globally, followed by Mexico (44.1%) and South Africa (3.9%) (McEachern, 2014; SAPPA, 2016). Furthermore, there is a strong interest in the South African pecan industry to export pecans particularly to China, because of the quality and high value of pecan nuts (Lemmer, 2020).

The ecological imbalance caused by biotic or abiotic factors across major production regions in South Africa have favoured the emergence of pathogenic microorganisms such as *Alternaria alternata* on pecans, thereby influencing the productivity and quality of pecan nuts (Achilonu et al., 2023c). *Alternaria alternata* has 9 to 11 chromosomes and a genome of up to 33.6 Mb (Akamatsu et al., 1999; Nguyen et al., 2016). It is a widespread ubiquitous endophytic fungus, as well as an aggressive and opportunistic plant pathogen (Lawrence et al., 2013; Woudenberg et al., 2013). The fungus produces grey to olive or olive brown woolly colonies (Simmons, 2007), with either single or long branched chains of brown and obclavate shaped conidia (Simmons, 1999). *Alternaria alternata* is known to primarily reproduce asexually (Thomma, 2003), and the developed conidia are mainly dispersed by air and rain splash across environments, or biotic activity such as human-mediated activities, insects, and animals (Woudenberg et al., 2015a).

*Alternaria alternata* causes black spot disease or Alternaria black spot (ABS) in plants (Kaur M. et al., 2020). Symptoms of ABS on plant tissues occur as dark brown circular lesions that enlarge, and eventually cause leaf death or rotten nuts (Achilonu et al., 2023c). The pathogen has been reported to cause ABS on crops such as citrus (Tran, 2018), shell ginger (Yan et al., 2022), cabbage (Rahimloo and Ghosta, 2015), persimmons (Kurt et al., 2010), pomegranates (Berbegal et al., 2014), pears (Terakami et al., 2016), sunflowers (Kgatle et al., 2018), and rubber trees (Cai et al., 2008). The fungus has also been shown to be responsible for black spot disease of nut trees such as hazelnuts and walnuts (Cheng et al., 2016). No research has been reported on the disease in pecans.

In South Africa, ABS is a common disease in pecan orchards, and it is caused by *A. alternata* (Achilonu et al., 2023c). In recent years, pecan farmers have stressed that the disease has become a growing problem, especially in major pecan production areas of the country. The importance of this pathogen in relation to the severity of disease has increased, contributing to an estimated yield loss of 25%–36% of the pecan nut production in South Africa, and ultimately the export capacity to China (Lemmer, 2020; Otto, 2021). Therefore, it is paramount to ease the risk of black spot disease by further studies.

A variety of registered fungicides are currently available and tested to control the attack of *A. alternata* against South African pecans (Achilonu et al., 2023a). Planting resistant pecan cultivars can be the most cost-effective and durable approach to manage this pathogen. The damage caused by the fungus, as well as the effectiveness of fungicide applications made to manage the ABS disease in pecans, appear inseparably linked to the ubiquitous and endophytic nature of *A. alternata* (Ding et al., 2019; Turzhanova et al., 2020). Usually, the rapid growth rate of asexual reproduction and short life cycle of *A. alternata* result in a lower ability to overcome the sensitivity to fungicides (Yang et al., 2019). Genetic structure and molecular mechanisms are needed to access the potential to alter this sensitivity and overcome resistance of the plant host (Drew et al., 2021; Sánchez-Torres, 2021). Hence it is important that these aspects be investigated for the ABS pathogen in the future.

Different molecular markers have been employed to identify genetic diversity of *A. alternata,* a pathogen causing diseases in various plants and vegetative crops. For example, simple-sequence repeats (SSRs), random amplified polymorphic DNA (RAPD) and inter-simple sequence repeat (ISSR) markers were successfully used to determine the genetic variability of *A. alternata* causing black spot in potato in China (Meng J. et al., 2015) and Iran (Nasr Esfahani, 2018). Restriction fragment length polymorphisms (RFLPs) outlined the distribution of *Alternaria* species causing early blight of potato (*Solanum tuberosum*) and tomato (*Solanum lycopersicum*) in Algeria and India (Ayad et al., 2019; Kaur T. et al., 2020). RFLPs was also employed to screen a large collection of *A. alternata* isolates causing black spot of pecan (*C. illinoinensis*) in South Africa without the need for expensive sequencing (Achilonu et al., 2023b). However, RFLP molecular markers have limited ability to discriminate between genetically similar isolates within *A. alternata* populations (Pryor and Michailides, 2002).

Random amplified microsatellites (RAMs) are based on Polymerase Chain Reaction (PCR) and is effective to detect high polymorphism levels for genetic diversity studies (Delgado-Alvarado et al., 2018). RAMs markers are co-dominant, fast, reproducible, and are also expressed throughout the fungal genome (Hantula et al., 1996; Hantula and Müller, 1997). The high frequencies of these microsatellites with motif makes RAMs ideal to resolve genetic variation of fungal populations (Sillo et al., 2016). Importantly, RAMs was able to precisely resolve the genetic diversity and population structure of pathogen populations, overcoming several limitations of gel electrophoresis and capillary-based methods (Luchi et al., 2020; Mugao, 2023). RAMs have been used previously to evaluate genetic diversity of other fungi, such as *Trichoderma* spp. (Siddiquee et al., 2012), and *Perenniporia fraxinea* (Sillo et al., 2016). RAMs markers (CCA_5_ and CGA_5_) have also been successfully used to assess the intraspecific genetic variation among 112 isolates of an *A. alternata* population from *Pinus tabulaeformis* in India (Ginoya and Gohel, 2016) and China (Guo et al., 2004).

No data have been generated regarding the genetic diversity of the ABS pathogen across the major pecan production regions in the Free State (FS), Eastern Cape (EC), Gauteng (GP), Kwazulu-Natal (KZN), Limpopo (LP), Northern Cape (NC), and North West (NW) provinces of South Africa. This hinders effective management of the pathogen. In the current study, RAMs was used to evaluate the genetic variation within and between *A. alternata* populations, sampled from pecan leaves, nuts-in-shucks, and shoots in the FS, EC, GP, KZN, LP, NC, and NW. This is to test the hypotheses that population subdivision exists among isolates of *A. alternata* based on geographic location. The information derived from this study could provide insight to the genetic diversity of this pathogen and help identify future genetic threats that could have a positive impact on the pathogenic ability of *A. alternata* on pecans.

## Material and methods

### Sampling locations and isolation of *Alternaria alternata*

**FIGURE 1:**
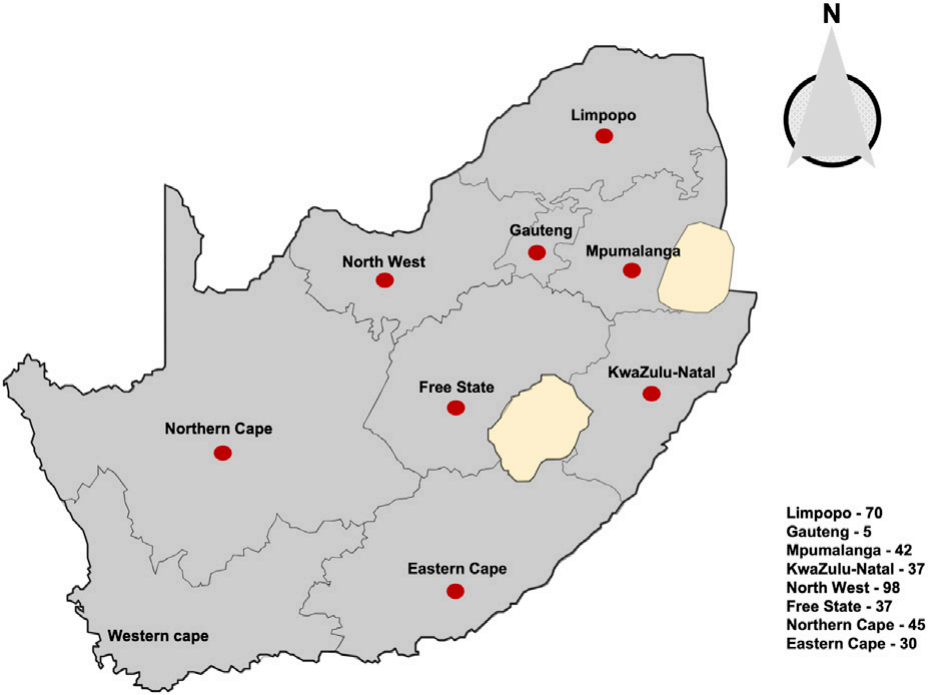
Map representing pecan plantations across eight provinces of South Africa where the 364 *Alternaria alternata* isolates was sampled, indicated by red dots. Numbers of isolates are indicated in the box, (see Supplementary Table S1 for detailed survey information).

Symptomatic and non-symptomatic pecan tissues such as nuts-in-shucks, leaves, and shoots were randomly collected in pecan orchards from eight major pecan geographical locations in South Africa between the 2017–2020 growing seasons (Figure 1). A total of 364 *A. alternata* isolates sampled from eight geographical locations were used in this study (Supplementary Table S1). Following the isolation procedures leading to single hyphal-tip cultures as described (Achilonu et al., 2023c), stock cultures were transferred into 2 mL cryogenic tubes (Merck Millipore, Pretoria, South Africa) containing 0.85% saline (NaCl)—15% glycerol solution and stored at −80°C until required.

### Random amplified microsatellites amplification and fragment analysis

Genomic (gDNA) was extracted from the newly isolated cultures using the ZR Quick DNA Fungal/Bacterial Microprep™Kit (Zymo Research, Johannesburg, South Africa). The DNA concentration and purity were determined with the NanoDrop Lite ND-2000 spectrophotometer (Thermo Fisher Scientific, Massachusetts, United States). The acquired gDNA purity for all the *A. alternata* isolates was ≥1.7 (A260 nm/A280 nm) (Sambrook et al., 2006), with the concentrations ranging between 10.0 and 802.8 ng/μL. Samples were diluted in sterile water to a concentration of 10 ng/μL, which was sufficient for RAMs molecular analysis (Delgado-Alvarado et al., 2018).

DNA samples were amplified in two separate PCR, using two modified labelled degenerate RAMs primers (CCA_5_ and CGA_5_) (Table 1) as described by Hantula et al. (1996) and these reactions were repeated twice. Each PCR amplification consisted of 50–100 ng of template DNA, 0.3 μM of each primer, 2.5 mM MgCl_2_, 0.3 mM of each dNTP, and 1 U KAPA HiFi HotStart DNA Polymerase (Kapa Biosystems-Roche, Basel, Switzerland), and ultra-pure dH_2_O to a final volume of 25 μL. The cycling conditions consisted of an initial denaturation step of 3 min at 95°C followed by 32 cycles of 20 s at 98°C, annealing 30 s at 56.3°C for CCA_5_ and 53.8°C for CGA_5_, extension at 72°C for 40 s, and a final extension step of 3 min at 72°C. The PCR reactions were performed in a T100TM Thermal Cycler (Bio-Rad, California, United States). For confirmation, RAMs amplicons were stained with GelRed nucleic acid stain (Thermo Fisher Scientific), separated by 5% agarose gels electrophoresed at 100 V—constant power, estimated using 1 Kb DNA ladder molecular weight marker, and visualised under UV light “Gel Doc EZ Gel Documentation System” (Bio-Rad).

**TABLE 1:** Random amplified microsatellites primers and annealing temperatures used to determine the genetic diversity of *Alternaria alternata* isolated from South African pecans (*Carya illinoinensis*).

5’ labelled phosphoramidite dye	Primers	Sequence (5’—3’)	Annealing temperature (°C)	Reference
6-FAM	CCA	DDB(CCA)_5_	56.3	Hantula et al. (1996)
VIC	CGA	DHB(CGA)_5_	53.8	

The following designations were used for the degenerated sites: H (A or T or C);

B (G or T or C); V (G or A or C); and D (G or A or T).

All the RAMs amplicons were pooled according to the biological sample, which resulted in one well per fungal isolate for the fragment analysis. Samples were run on an ABI 3500xl Genetic Analyser (Applied Biosystems), using standard protocols. The Genetic Analyser used matrix standard dye set (ROX^TM^) to analyse amplicons amplified with RAMs primers labelled with fluorescent dyes, namely, 6-FAM and VIC (Thermo Fisher Scientific). The total reaction volume of 10 μL consisted of 8.6 μL Hi-Di™ formamide (Thermo Fisher Scientific) mixed with 0.4 μL GeneScan™2500 ROX™ and 1 μL of PCR product. Only the fragments shorter than 1,060 base pairs (bp) were considered reproducible because of the limited fragment size (±1,100 bp) of the Genetic Analyser capillary. Data containing the alleles (Supplementary Table S2) were manually scored for each primer using GeneMapper v1.6 software (Applied Biosystems).

### Data analysis

GenAlEx v6.5 software was used to evaluate the genetic diversity within the 8 *A. alternata* populations (Peakall and Smouse, 2012). Each fragment was distinguished according to the base pair size (bp) and thus, considered as a single molecular character. The amplicon profiles for each sample-primer combination were assessed and used to construct a binary matrix coded by 1 (presence) or 0 (absence) with all individual samples per amplified loci. The genetic variability of the 364 *A. alternata* isolates for both individual and combined primers (binary haploid data) was determined with the following parameters: Alleles: *Na* (number of average alleles), *Ne* (number of effective alleles), *I* (Shannon’s information index), *H* (observed diversity), *Uh* (unbiased expected diversity), and *PPL* (percentage of polymorphic loci) by using GenAlEx v6.5 software. Notably, identical samples of individuals were identified using the probability identity matrices.

Hierarchical analysis of molecular variance (AMOVA) was evaluated using GenAlEx v6.5 (Peakall and Smouse, 2012). The analysis provided an alternative clustering approach to view the genetic similarity and diversity of isolates among and within the eight populations. Principal coordinate analysis (PCoA) was conducted based on genetic distance (*GD*) for the combined data by covariance-standardised method as implemented using the same statistical package. Genetic structure for the eight populations was analysed using STRUCTURE v2.3.4 (Pritchard et al., 2000). *K*-values from 2 to 8 populations were tested, at least 10 simulations were executed by each *K*-value, after which the burn-in length was set to 1,000,000, and the number of MCMC repetitions after burn-in at 500,000. The result files from the runs were uploaded to online STRUCTUREHARVESTER, and then the delta *K* (*ΔK ad hoc*) method described by Evanno et al. (2005) was used to detect the most probable number of genetic clusters (*K*). The genetic relationships between isolates were further estimated by submitting the combined data set to a cluster analysis (Ward´s method) using Past 4.05 package (Hammer et al., 2001).

## Results

### Random amplified microsatellites amplification and fragment analysis

**FIGURE 2:**
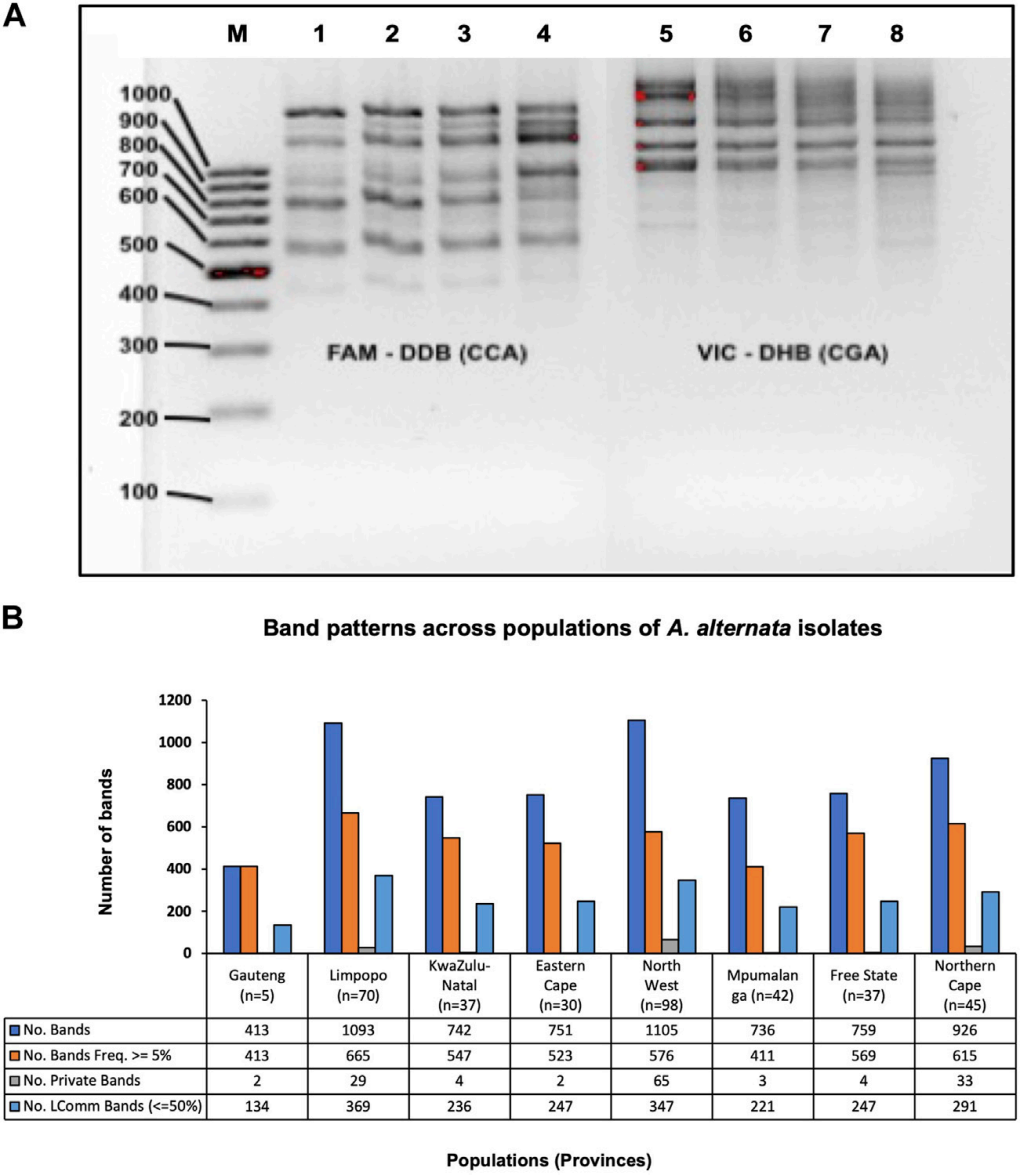
**(A)** An example of RAMs agarose gel (5%) depicting visible bands of *A. alternata* isolates. Lanes left to right: M = molecular weight marker of 100 bp ladder (Thermo Fisher Scientific, Massachusetts, USA), Lane 1–4 = amplicons with RAMs marker (CCA5), Lane 5–8 = amplicons with RAMs marker (CGA5). **(B)** Combined amplified RAMs markers (CCA5 and CGA5) band patterns across 364 *A. alternata* isolates from 8 populations. No. bands = No. of total bands (6525), No. Bands Freq. ≥5% = No. of different bands (4319) with a Frequency ≥5%, No. Private Bands = No. of bands (142) unique to a single population, No. LComm Bands (≤ 50%) = No. of locally common bands (2092) Freq. ≥5%) found in 50% or fewer populations. Abbreviation: n = number of *A. alternata* isolates, No. = number.

Agarose gel images for both primers showed visible banding patterns from 450 to 1,500 bp (Figure 2A). More fragment sizes were generated with fragment analysis by GeneMapper v3, between 13 bp and 1,060 bp for CCA_5_ primer and 15 and 1,060 bp for CGA_5_ primer (data not shown). The result of the band patterns of the combined primers across populations revealed that there were 4,319 (66%) polymorphic bands, 142 (2%) bands were unique to a single population and 2092 (32%) were monomorphic bands (Freq. >= 5%) found in 50% or fewer populations (Figure 2B).

### Genetic variation

The results showed various levels of genetic diversity indices of *A. alternata* in all sub-populations (Table 2). The two RAMs primers amplified a total of 6,525 alleles, with 3,182 alleles for the CGA_5_ primer and 3,343 alleles for the CCA_5_ primer. The combined primer analysis showed that the parentage of polymorphism within population varied from 28.0% to 80.6% in the Gauteng and North-West populations, respectively. The number of different alleles ranged from 0.58 (GP) to 1.61 (NW), and the number of effective alleles ranged between 1.16 (NW and MP) and 1.23 (NC). The Shannon’s information diversity index (*I*) ranged from 0.16 (GP) to (LP and NC), with average at 0.20. Nei’s observed genetic diversity (*H*) ranged between 0.11 (GP, EC, and MP) and 0.15 (NC). Nei’s unbiased genetic diversity (*Uh*) ranged from 0.11 (MP) to 0.15 (LP and NC). The mean expected diversity for the total of isolates was 0.13, indicating about 13% were expected to be heterozygous at a given locus. There was a slight increase in the mean value of Nei’s unbiased estimate of expected genetic diversity (*Uh*), contrary to the observed diversity (*H*) across both individual primers and combined for each population.

**TABLE 2:** Population’s estimation of *Alternaria alternata* isolates based on number of different alleles, polymorphism (%), and diversity level.

Population/Province (abbreviation)	CCA primer							CGA primer								Combined (CCA and CGA) primer						
	*N*_*T*_	*PP* (%)	*Na*	*Ne*	*I*	*H*	*Uh*	*N*_*T*_	*PP* (%)	*Na*	*Ne*	*I*	*H*	*Uh*	*N*_*T*_		*PP* (%)	*Na*	*Ne*	*I*	*H*	*Uh*
Gauteng (GP)	221	30.3	0.62	1.20	0.17	0.12	0.14	192	25.6	0.54	1.18	0.15	0.10	0.13	413		28.0	0.58	1.19	0.16	0.11	0.14
Limpopo (LP)	573	81.5	1.63	1.20	0.24	0.14	0.14	520	77.8	1.56	1.23	0.25	0.15	0.15	1,093		79.7	1.59	1.22	0.24	0.14	0.15
KwaZulu-Natal (KZN)	432	61.5	1.23	1.20	0.21	0.13	0.13	310	46.4	0.93	1.16	0.17	0.10	0.11	742		54.0	1.08	1.18	0.19	0.12	0.12
Eastern Cape (EC)	399	56.8	1.14	1.17	0.19	0.12	0.12	352	52.7	1.05	1.16	0.18	0.11	0.11	751		54.8	1.10	1.17	0.19	0.11	0.12
North West (NW)	577	82.1	1.64	1.16	0.20	0.12	0.12	528	79.0	1.58	1.16	0.20	0.12	0.12	1,105		80.6	1.61	1.16	0.20	0.12	0.12
Mpumalanga (MP)	354	50.4	1.01	1.16	0.17	0.10	0.10	382	57.2	1.14	1.17	0.18	0.11	0.11	736		53.8	1.07	1.16	0.17	0.11	0.11
Free State (FS)	360	51.2	1.02	1.18	0.18	0.11	0.12	399	59.7	1.20	1.20	0.21	0.13	0.13	759		55.5	1.11	1.19	0.20	0.12	0.12
Northern Cape (NC)	427	60.7	1.22	1.21	0.22	0.14	0.14	499	74.7	1.49	1.26	0.27	0.17	0.17	926		67.7	1.35	1.23	0.24	0.15	0.15
Mean	-	59.3	1.19	1.19	0.20	0.12	0.13	-	59.1	1.19	1.19	0.20	0.12	0.13	-		59.3	1.19	1.19	0.20	0.12	0.13
SE	-	5.99	0.12	0.01	0.01	0.01	0.01	-	6.43	0.13	0.01	0.01	0.01	0.01	-		6.00	0.12	0.01	0.01	0.01	0.01
Total	3,343							3,182							6,525							

*N*_*T*_: total number of alleles.

*PP*(%): Polymorphic percentage.

*Na*: Number of different alleles.

*Ne*: Number of effective alleles.

*I*: Shannon’s information index.

*H*: observed diversity.

*Uh*: Unbiased expected diversity.

The analysis of molecular variance (AMOVA) showed that there was a significant difference (*p* < 0.001) within and among *A. alternata* populations (Table 3). The results showed that 88% of the genetic variation was found within populations with no significant (*p* > 0.001) genetic differentiation (*Nm* = 3.59, PhiPT = 0.12). A 12% variation was observed among populations with significant (*p* < 0.001) genetic differentiation (*Nm* = 2.89, PhiPT = 0.08). The estimated gene flow value of 3.59 and 2.89 indicated high gene flow of *A. alternata* within and among populations, respectively. Nm is the product of population size and the proportion of the number of alleles exchanged between populations per generation (Whitlock and Mccauley, 1999). The mean PhiPT value of 0.12 and 0.14, indicated low genetic differentiation within and among populations, respectively. PhiPT represents the analogous standardised measures of the degree of genetic differentiation of within and among populations, indicating scores for both measures range from 0 to 1 that equals no differentiation (Peakall and Smouse, 2012).

**TABLE 3:** Summary of analysis of molecular variance (AMOVA) showing the genetic variation within and among populations of 364 *Alternaria alternata* isolates from South African pecans (*Carya illinoinensis*).

Sources of variation	df	SS	MS	Est. Var.	% Of variation	Nm (haploid)	PhiPT	*p*-value
Among populations	7	2,163.77	309.11	6.10	12	2.86	0.14	*p* > 0.001
Within populations	357	15,647.81	43.83	43.83	88	3.59	0.12	*p* < 0.001
Total	364	17,811.58		49.93	100			

df: Degrees of freedom.

SS: sum of squares.

MS: mean square.

Est. Var.: estimated variance.

*Nm*: Haploid Number of migrants.

Probability, P (rand >= data).

PhiPT: proportion of the total genetic variance that is due to the variance within populations is based.

PhiPT = AP/(WP + AP) = AP/TOT.

Nm (Haploid) = [(1/PhiPT) - 1]/2.

Key: AP, Est. Var. among pops, WP, Est. Var. within populations.

*p*-value is based on 999 permutations.

**FIGURE 3:**
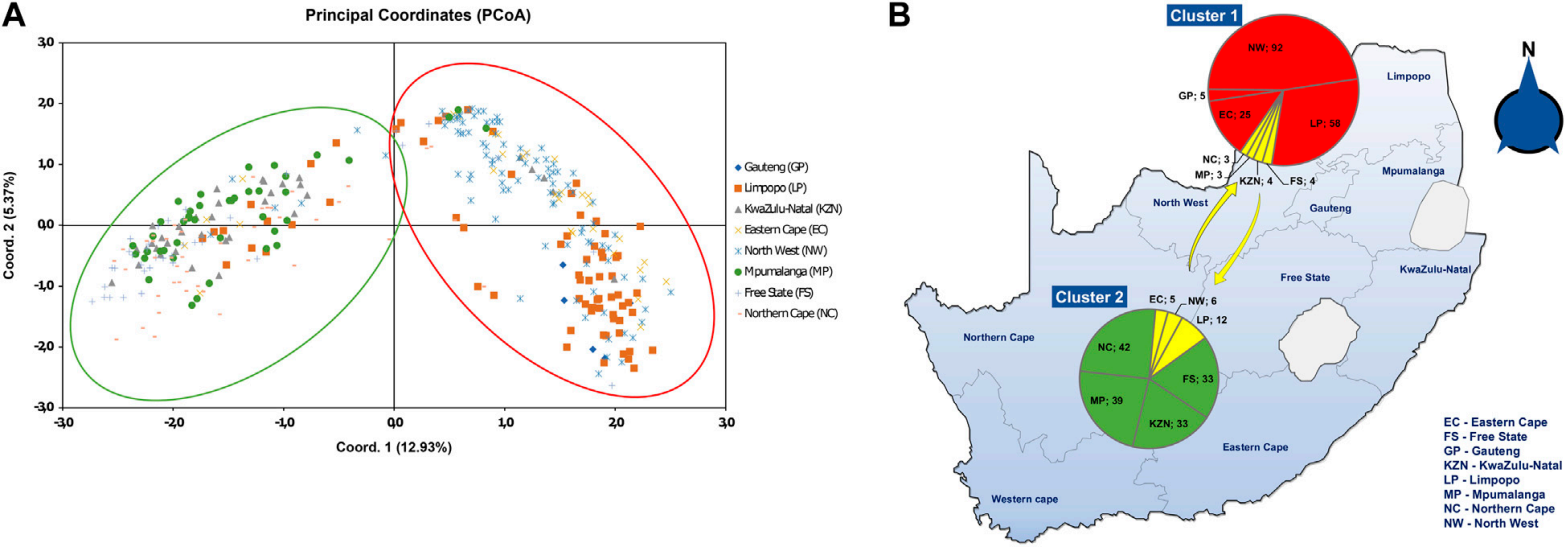
**(A)** Principal coordinate analysis (PCoA) plot of 364 *A. alternata* isolates from eight populations, based on RAMs markers (CCA_5_ and CGA_5_) and the genetic distance matrix for haploid distance using GenAlEx. The scatter plot shows the first and second principal coordinates that best explain the diversity in the population. Cluster 1 consists mostly fungal isolates from Eastern Cape, Gauteng, North-West and Limpopo, with few isolates from Northern Cape, Mpumalanga, KwaZulu-Natal, and Free State. Cluster 2 depicts isolates mostly from Mpumalanga, KwaZulu-Natal, Free State and Northern Cape, with few representative isolates from Eastern Cape, North-West and Limpopo. **(B)** Schematic map distribution of the *A. alternata* isolates showing presence of the two clusters as area in pie graphs. Red area in cluster 1 and green area in cluster 2 shows most isolates per population, and yellow depicts some isolate overlap.

The Principal Coordinate analysis (PCoA) (Figure 3A) depicted a cumulative 12.93% variation between populations that grouped into two clusters of the first three axes. Cluster 1 contained fungal isolates from EC, GP, NW, and LP, with few isolates from FS, MP, NC and KZN. Cluster 2 was formed by isolates from MP, KZN, FS and NC with few representative isolates from EC, NW, and LP. The PCoA analysis also showed that a small number of fungal isolates from Limpopo, North-West, and Eastern Cape were distributed in all the four coordinates. A schematic map shows the distributions of the *A. alternata* genotypes (Figure 3B), where the pie charts contained genotypes from each cluster. Cluster 1 (red) contained genotypes from NW (92), GP (5), EC (25), and LP (58). Cluster 2 (green) had genotypes from NC (42), MP (39), KZN (33), and FS (33), while yellow indicated genotypes overlapping the two clusters.

### Population structure and *Alternaria alternata* dendrogram clusters

**FIGURE 4:**
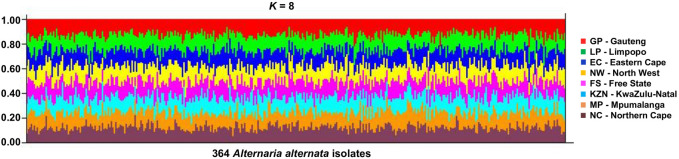
Structure output for the various possible values of *K* = 8 for the 364 *A. alternata* isolates from eight populations—Free State (FS), Eastern Cape (EC), Gauteng (GP), Kwazulu-Natal (KZN), Limpopo (LP), Northern Cape (NC), and North-West (NW) province (South Africa). Each vertical line represents one individual genotype, and each shade of colours refer to the eight populations. The proportion of each colour segment indicates the likelihood of an individual being assigned to the population represented by that colour.

STRUCTURE showed no even distribution of genotypes and thus, no distinct geographic population structure was observed in the data based on the STRUCTURE HARVESTER output (Supplementary Figure S1), with the most likely *K* value as *K* = 8 (Figure 4). Each coloured bar represented an individual isolate of the *Alternaria* population. The isolates from the same collection location had almost a similar level of admixture. Notably, though, Δ*K* cannot find the true number of populations if there is less variation of the fungal, i.e., if *K* = 1. However, none of the multiple STRUCTURE analyses contradicted the finding that *K* = 1.

**FIGURE 5:**
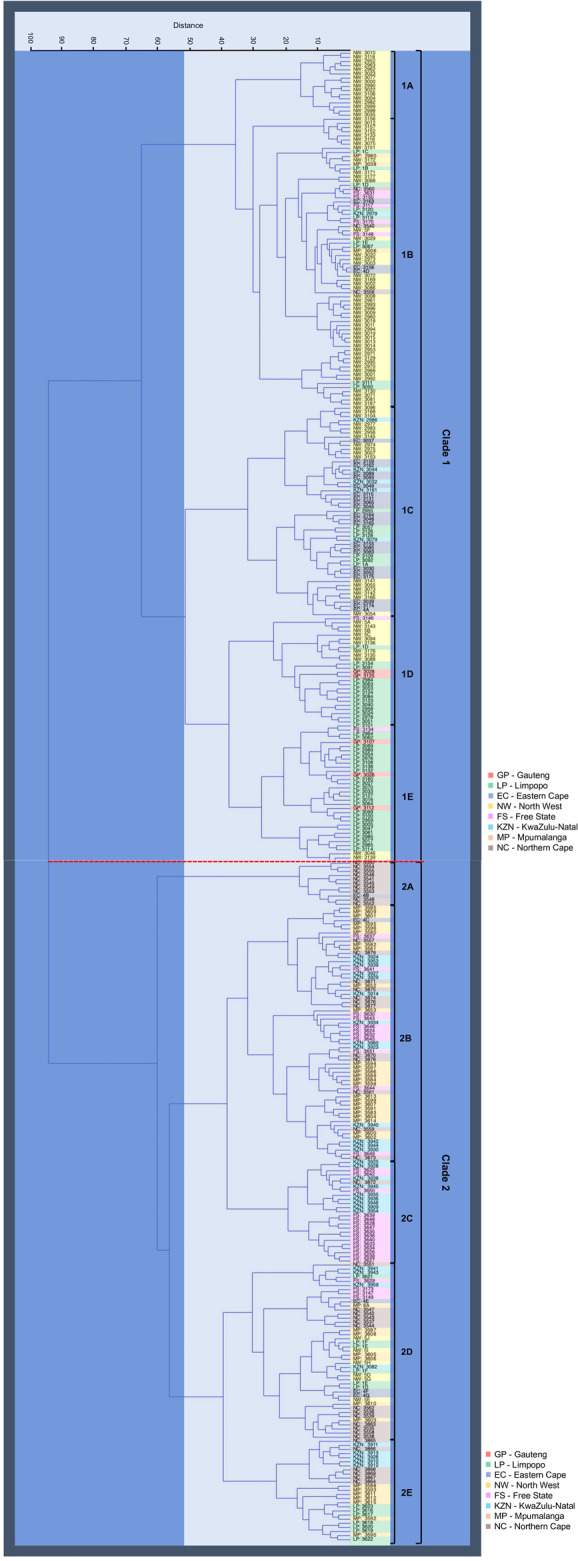
Hierarchical dendrogram cluster analysis (Euclidean distance and Ward Group linkage Method) for the 364 *A. alternata* isolates from eight populations - Free State (FS), Eastern Cape (EC), Gauteng (GP), Kwazulu-Natal (KZN), Limpopo (LP), Northern Cape (NC), and North-West (NW) province (South Africa), annotated by different colours in block. The analysis unveiled two main distinct clades (clade 1 and 2) and 5 subclades (1A—1E and 2A—2E) was formed on the respective two clades.

The dendrogram cluster tree generated by Ward’s analysis unveiled two main distinct clades consisting of 10 subclades (Figure 5) that revealed similar findings as those of PCoA analysis clusters. Clade 1 consists of 198 *A. alternata* isolates, with majority isolates from GP (5), EC (25), LP (57), and NW (92). A small number of isolates from MP (3), NC (3), KZN (6), and FS (7) were found in the clade. Five subclades (1A, 1B, 1C, 1D, and 1E) were formed within clade 1. Subclade “1A” contained 16 isolates from NW, while subclade “1B” had 70 isolates distributed amongst LP (9), MP (3), NC (3), EC (3), KZN (1), and NW (46). Subclade “1C” consisted of 51 isolates from KZN (5), EC (22), LP (7), and NW (17). Twenty-six *A. alternata* isolates from FS (1), LP (14), GP (2), and NW (9) were grouped in subclade “1D”, and sub-cluster “1E” had 35 isolates from FS (1), GP (3), NW (4), and LP (27).

Clade 2 had a cluster pattern similar to clade 1, comprised of 166 isolates, the majority of which came from FS (30), KZN (31), MP (39), and NC (42) and few isolates from EC (5), NW (6), and LP (13). Five subclades were distinguished (2A, 2B, 2C, 2D, and 2E). Subclade “2A” had 11 isolates from EC (1), and NC (10), subclade “2B” consisted of 62 isolates from EC (1), FS (11), NC (12), KZN (13), and MP (25), and subclade “2C” had 25 isolates shared between NC (1), KZN (9), and FS (15). Subclade “2D” had 43 *A. alternata* isolates between EC (3), FS (4), KZN (4), NW (5), LP (6), MP (7), NC (14), and subclade “2E” included 25 isolates from KZN (5), NC (6), MP (7), and LP (7).

## Discussion

The present study was conducted to evaluate the genetic diversity and population structure in *A. alternata* populations sampled from South African pecans (*C. illinoinensis*) in the FS, EC, GP, KZN, LP, NC, and NW. The study is the first to document the use of RAMs primers to evaluate the genetic diversity of *A. alternata* isolates causing black spot disease on pecans in South Africa. The analyses showed low genetic diversity in all *A. alternata* populations. There were no major dominant genotypes present across all provincial geographical areas, but two major groups from the Northern and Southern parts of South Africa were formed. This fungus has low genetic variation, which has positive implications for the efficacy of fungicides and reduced genetic fitness due to the low pathogen variability.

RAMs proved valuable to study the genetic diversity and population structure of *A. alternata*. In this study, RAMs assisted to produce a high number of 6,525 alleles from the 364 *A. alternata* isolates across the eight populations. The percentage polymorphism of the number of alleles was above average and effective alleles. The number of alleles obtained exceeded the only previous study reported to describe the genetic diversity (*H* = 2.79) of 112 *A. alternata* isolates from *Pinus tabuliformis* in China (Guo et al., 2004), with 1,273 alleles detected using the same two RAMS primers as in this study. This could be due to the poor band resolution produced from agarose gel method the authors used (Barril and Nates, 2012), and are not suitable for amplified samples with low molecular weight (Akbari et al., 2008).

The analysis of genetic variability across all loci confirmed low genetic diversity within and among *A. alternata* populations. This is shown by a low mean percentage of polymorphic loci (59.3), number of observed alleles (1.19), number of effective alleles (1.19), Shannon’s information index (0.20), Observed diversity (0.12), and Unbiased expected diversity (0.13) across the eight populations. Similar, but generally lower numbers. Similar levels (*H* = 0.11 and 0.16) of genetic diversity was found by Weir et al. (1998), where the authors used RAPDs technique to evaluate the genetic variation of predominantly United States isolates of *A. solani* and *A. alternata,* known to cause early blight in potato (*S. tuberosum*) and tomato (*S. lycopersicum*). In South Africa, van der Waals et al. (2004) reported a mean genetic diversity of 0.20 for *A. solani* isolates from potatoes (*S. tuberosum*) using similar RAMS primer combinations. In contrast, relatively high genetic diversity values (*H* = 0.952, 0.923, and 0.916) were reported for *A. alternata* populations across the mainland in the United States (Woudenberg et al., 2015a).

The AMOVA and STRUCTURE analyses demonstrated the genetic differentiation and population structure of *A. alternata* populations. The AMOVA accounted for a high level of genetic variation within populations, and low variability among populations. The mean genetic differentiation among *A. alternata* populations indicated a weak and nonsignificant genetic differentiation existing within the populations. Our finding based on the significant genetic differentiation of *A. alternata* populations was similar and consistent with previous studies on genetic diversity of *Alternaria* species (Turzhanova et al., 2020). Gene flow (*Nm*) for the combined dataset also explains the level of genetic variability in the populations. High level of gene flow can only indicate a more homogeneous population structure and a lower degree of genetic differentiation among populations of *A. tenuissima* from wheat kernels in Russia (Gannibal et al., 2007). The high average gene flow (*Nm* = 3.59) within populations and among populations (*Nm* = 2.86) in the present study can be associated to the low genetic differentiation. Similar to those reported in other studies by (Meng J. W. et al., 2015; Yang et al., 2018), showed *Nm* values of 5.80 and 3.70, respectively. Low genetic differentiation and the lack of shape between certain populations (Garant et al., 2007; Stefansson et al., 2014) was supported by the results of STRUCTURE analyses. The analysis weakly inferred eight sub-groups (*K* = 8), where all individual isolates tested share genetic information (alleles) inherited from the sub-groups. Thus, this corroborates the presence of high potential of genetic admixture and closed relationships among isolates of the different sampling pecan sites as a result of high gene flow. In accordance with STRUCTURE analyses in this study, no population substructure in *A. alternata* populations were found from wheat (*Triticum aestivum*) in Kazakhstan (Turzhanova et al., 2020), and from (*Citrus* spp.) in Morocco (Zelmat et al., 2021).

The PCoA and UPGMA cluster analyses did not sharply group the *A. alternata* populations according to their geographical origins and the clustering pattern was weak enough to support the concept of “isolation by distance”. Our findings showed two main clusters of similar haplotypes, with each cluster containing the majority of isolates from four different populations. It was evident that both analyses depicted no distinct relationship between the fungal isolates and their geographic locations. The current results support previous studies (Yang et al., 2018), revealing that *A. alternata* isolates were not clustered based on their locations.

These overall findings have implications for the management of ABS on South African pecans. Firstly, asexually reproducing populations of *A. alternata* could account for low genetic diversity and random association between different loci (Woudenberg et al., 2015b; Kreis et al., 2016; Ozkilinc and Sevinc, 2018). A proposed explanation in this study was that asexual populations can reproduce many spores, the spore spreads, and high levels of clonality are dominated by few genotypes and low gene diversity, therefore promotes the low level of genetic variation within populations of *A. alternata*. In addition, other forms of recombination could also take place. For instance, a study of *A. alternata* populations, causing early blight in potato (*S. tuberosum*) in China revealed two subpopulations that showed the ability to recombine through many parasexual cycles of asexual propagation with fewer cycles of cryptic sexual reproduction (Meng J. W. et al., 2015), based on the evolving loci and the presence of both mating-types (*MAT1-1* and *MAT1-2*).

Another explanation for the random association of alleles in *A. alternata* can be ascribed to the nature of microsatellites. For example, Buschiazzo and Gemmell (2006) proposed that these evolving loci are altered through birth-and-death evolution such that they are highly polymorphic in eukaryotes. It is likely that over lengthy periods of asexual reproduction, a microsatellite locus can hyper-mutate in large populations such as those usually found in *A. alternata* (Woudenberg et al., 2015b). If this is equally applicable on every microsatellite, such events could explain the high number of evolving alleles found in the present study. The aforementioned results accounts for two possibilities: either the size limitations on microsatellite loci led to a very high probability of size homoplasy, which confuses the detection of hyper-mutation (Angers et al., 2000; Buschiazzo and Gemmell, 2006) or *A. alternata* isolates has been propagating asexually for such a long time that loci are hyper-mutated. Thus, future studies should perhaps look at more than one approach.

Large quantities of fungal spores can be spread mostly through wind, rain, and human-mediated dispersal (Meng et al., 2018; Yang et al., 2018). South Africa has some heavy winds and rain, which could result in high spread of *A. alternata* within the populations. Also, the isolates in this study were sampled from eight geographic regions, separated by more than 800 km from each other (www.google.com/maps/dir/), which makes it difficult for *A. alternata* spores to be disseminated through wind for such a distance. Long-distance movement of spores can most likely be attributed to human-mediated gene flow, which has been reported in *A. alternata* populations in potato growing areas in China (Meng et al., 2018). How pecan farmers in South Africa obtained and move their grafted seedling plants from nurseries within and across different locations could also account for the distribution of genotypes. Most pecan nurseries still use self-seeding plants and grafting for cultivation (Krüger, 2016) and *A. alternata* could already be present in the plant tissue in nurseries. Another possibility could be that the diversity of genotypes was already present in the environment from different plant hosts, soils, and decaying organic material. This then acts as a reservoir of inoculum to infect pecans, especially newly planted seedlings, or planting of trees in virgin land.

## Conclusion

This study laid the groundwork for future studies on the population of *A. alternata* across pecan orchards in South Africa. Low genetic diversity was established in *A. alternata* populations, brought about by the high gene flow within and among the populations. This may explain the non-correlation with the geographic distribution of the *A. alternata* pathogen, which is predominantly isolated in the eight major pecan producing provinces. Cautious intervention could be implemented by pecan farmers when applying different fungicides that can specifically inhibit the growth of this fungus. Finally, the production of pathogen free pecan trees in nurseries, whether through resistance breeding or careful selection of plant material, could improve the control of ABS, since pathogen populations with high evolutionary potential are more likely to overcome host genetic resistance (McDonald and Linde, 2002).

## Data Availability

The original contributions presented in the study are included in the article/Supplementary Material, further inquiries can be directed to the corresponding author.
