# Supplementary material for: Random amplified microsatellites (RAMS) analysis ascertains genetic variation of Alternaria alternata causing black spot disease on Carya illinoinensis in South Africa
Source: Front Genet. 2023 Sep 27;14:1213102. doi: 10.3389/fgene.2023.1213102 (PMC10569608; doi:10.3389/fgene.2023.1213102)
Supplement: Supplementary file 1 [file Table1.docx]

**Supplementary Table S1** *Alternaria alternata* isolates used in this study with the locality and substrates (2017 -2020)

| **Number of Samples** | **Isolates Number** | **Location** | **Coordinates** | **Pecan substrates** |
| --- | --- | --- | --- | --- |
| 1 | CGJM 3112 | Gauteng | 25°35'13.6"S 28°33'31.6"E | Symptomatic Nuts |
| 2 | CGJM 3028 | Gauteng | 25°35'13.6"S 28°33'31.6"E | Symptomatic Leaves |
| 3 | CGJM 3026 | Gauteng | 25°35'13.6"S 28°33'31.6"E | Symptomatic Leaves |
| 4 | CGJM 3136 | Gauteng | 25°35'13.6"S 28°33'31.6"E | Symptomatic Shoots |
| 5 | CGJM 3097 | Gauteng | 25°35'13.6"S 28°33'31.6"E | Symptomatic Leaves |
| 6 | CGJM 3053 | Limpopo | 24°40'40.5"S 28°29'55.6"E | Symptomatic Nuts |
| 7 | CGJM 3119 | Limpopo | 24°40'40.5"S 28°29'55.6"E | Symptomatic Shoots |
| 8 | CGJM 3057 | Limpopo | 24°40'40.5"S 28°29'55.6"E | Symptomatic Shoots |
| 9 | CGJM 2955 | Limpopo | 24°40'40.5"S 28°29'55.6"E | Symptomatic Shoots |
| 10 | CGJM 3109 | Limpopo | 24°40'40.5"S 28°29'55.6"E | Symptomatic Leaves |
| 11 | CGJM 3126 | Limpopo | 24°40'40.5"S 28°29'55.6"E | Symptomatic Leaves |
| 12 | CGJM 3120 | Limpopo | 24°40'40.5"S 28°29'55.6"E | Symptomatic Leaves |
| 13 | CGJM 3128 | Limpopo | 24°40'40.5"S 28°29'55.6"E | Symptomatic Leaves |
| 14 | CGJM 3154 | Limpopo | 24°40'40.5"S 28°29'55.6"E | Symptomatic Leaves |
| 15 | CGJM 3067 | Limpopo | 24°40'40.5"S 28°29'55.6"E | Symptomatic Leaves |
| 16 | CGJM 3091 | Limpopo | 24°40'40.5"S 28°29'55.6"E | Symptomatic Leaves |
| 17 | CGJM 3084 | Limpopo | 24°40'40.5"S 28°29'55.6"E | Symptomatic Leaves |
| 18 | CGJM 3160 | Limpopo | 24°40'40.5"S 28°29'55.6"E | Symptomatic Leaves |
| 19 | CGJM 3123 | Limpopo | 24°40'40.5"S 28°29'55.6"E | Symptomatic Leaves |
| 20 | CGJM 3124 | Limpopo | S 23 05'12.1'' E 030 10'50.0'' | Symptomatic Leaves |
| 21 | CGJM 3064 | Limpopo | S 23 05'12.1'' E 030 10'50.0'' | Symptomatic Leaves |
| 22 | CGJM 3070 | Limpopo | S 23 05'12.1'' E 030 10'50.0'' | Symptomatic Leaves |
| 23 | CGJM 3061 | Limpopo | S 23 05'06.3 E 030 11 05.6" | Symptomatic Leaves |
| 24 | CGJM 3017 | Limpopo | S 23 05'06.3 E 030 11 05.6" | Symptomatic Leaves |
| 25 | CGJM 2985 | Limpopo | S 23 05'06.3 E 030 11 05.6" | Symptomatic Leaves |
| 26 | CGJM 3005 | Limpopo | S 23 05'06.3 E 030 11 05.6" | Symptomatic Leaves |
| 27 | CGJM 3047 | Limpopo | 24°25'42.1"S 28°35'41.0"E | Symptomatic Shoots |
| 28 | CGJM 3111 | Limpopo | 24°25'42.1"S 28°35'41.0"E | Symptomatic Shoots |
| 29 | CGJM 2965 | Limpopo | 24°25'42.1"S 28°35'41.0"E | Symptomatic Leaves |
| 30 | CGJM 3114 | Limpopo | 24°25'42.1"S 28°35'41.0"E | Symptomatic Leaves |
| 31 | CGJM 3101 | Limpopo | 24°25'42.1"S 28°35'41.0"E | Symptomatic Leaves |
| 32 | CGJM 3040 | Limpopo | 24°25'42.1"S 28°35'41.0"E | Symptomatic Leaves |
| 33 | CGJM 2978 | Limpopo | 24°25'42.1"S 28°35'41.0"E | Symptomatic Leaves |
| 34 | CGJM 3051 | Limpopo | 24°25'42.1"S 28°35'41.0"E | Symptomatic Leaves |
| 35 | CGJM 3024 | Limpopo | 24°25'42.1"S 28°35'41.0"E | Symptomatic Leaves |
| 36 | CGJM 3050 | Limpopo | 24°25'42.1"S 28°35'41.0"E | Symptomatic Leaves |
| 37 | CGJM 3100 | Limpopo | 24°25'42.1"S 28°35'41.0"E | Symptomatic Leaves |
| 38 | CGJM 3099 | Limpopo | 24°25'42.1"S 28°35'41.0"E | Symptomatic Leaves |
| 39 | CGJM 2959 | Limpopo | 24°25'42.1"S 28°35'41.0"E | Symptomatic Leaves |
| 40 | CGJM 2958 | Limpopo | 24°25'42.1"S 28°35'41.0"E | Symptomatic Leaves |
| 41 | CGJM 3027 | Limpopo | 24°25'42.1"S 28°35'41.0"E | Symptomatic Leaves |
| 42 | CGJM 3108 | Limpopo | 29°36'59.8"S 30°27'53.6"E | Symptomatic Nuts |
| 43 | CGJM 3132 | Limpopo | 29°36'59.8"S 30°27'53.6"E | Symptomatic Shoots |
| 44 | CGJM 3138 | Limpopo | 29°36'59.8"S 30°27'53.6"E | Symptomatic Leaves |
| **Supplementary**  **Table S1:** (continued) |  |  |  |  |
| **Number of Samples** | **Isolates Number** | **Location** | **Coordinates** | **Pecan substrates** |
| 45 | CGJM 2954 | Limpopo | 29°36'59.8"S 30°27'53.6"E | Symptomatic Leaves |
| 46 | CGJM 2989 | Limpopo | 29°36'59.8"S 30°27'53.6"E | Symptomatic Leaves |
| 47 | CGJM 3069 | Limpopo | 29°36'59.8"S 30°27'53.6"E | Symptomatic Leaves |
| 48 | CGJM 2964 | Limpopo | 29°36'59.8"S 30°27'53.6"E | Symptomatic Leaves |
| 49 | CGJM 2984 | Limpopo | 29°36'59.8"S 30°27'53.6"E | Symptomatic Leaves |
| 50 | CGJM 3062 | Limpopo | 29°36'59.8"S 30°27'53.6"E | Symptomatic Leaves |
| 51 | CGJM 3063 | Limpopo | 29°36'59.8"S 30°27'53.6"E | Symptomatic Leaves |
| 52 | CGJM 3033 | Limpopo | 29°36'59.8"S 30°27'53.6"E | Symptomatic Leaves |
| 53 | CGJM 3127 | Limpopo | 29°36'59.8"S 30°27'53.6"E | Symptomatic Leaves |
| 54 | CGJM 2976 | Limpopo | 29°36'59.8"S 30°27'53.6"E | Symptomatic Leaves |
| 55 | CGJM 3016 | Limpopo | 29°36'59.8"S 30°27'53.6"E | Symptomatic Leaves |
| 56 | CGJM 3092 | Limpopo | 29°36'59.8"S 30°27'53.6"E | Symptomatic Leaves |
| 57 | CGJM 1A | Limpopo | 29°36'59.8"S 30°27'53.6"E | Symptomatic Leaves |
| 58 | CGJM 1B | Limpopo | 29°36'59.8"S 30°27'53.6"E | Symptomatic Leaves |
| 59 | CGJM 1C | Limpopo | 24°40'40.5"S 28°29'55.6"E | Symptomatic Shoots |
| 60 | CGJM 1D | Limpopo | 24°25'42.1"S 28°35'41.0"E | Symptomatic Leaves |
| 61 | CGJM 1E | Limpopo | 24°25'42.1"S 28°35'41.0"E | Symptomatic Leaves |
| 62 | CGJM 1F | Limpopo | 29°36'59.8"S 30°27'53.6"E | Symptomatic Leaves |
| 63 | CGJM 1D | Limpopo | 29°36'59.8"S 30°27'53.6"E | Symptomatic Leaves |
| 64 | CGJM 1E | Limpopo | 24°40'40.5"S 28°29'55.6"E | Symptomatic Shoots |
| 65 | CGJM 1F | Limpopo | 24°25'42.1"S 28°35'41.0"E | Symptomatic Leaves |
| 66 | CGJM 1D | Limpopo | 24°25'42.1"S 28°35'41.0"E | Symptomatic Leaves |
| 67 | CGJM 1E | Limpopo | 29°36'59.8"S 30°27'53.6"E | Symptomatic Leaves |
| 68 | CGJM 3616 | Limpopo | 29°36'59.8"S 30°27'53.6"E | Symptomatic Leaves |
| 69 | CGJM 3617 | Limpopo | 29°36'59.8"S 30°27'53.6"E | Symptomatic Leaves |
| 70 | CGJM 3618 | Limpopo | 29°36'59.8"S 30°27'53.6"E | Symptomatic Leaves |
| 71 | CGJM 3619 | Limpopo | 29°36'59.8"S 30°27'53.6"E | Symptomatic Leaves |
| 72 | CGJM 3620 | Limpopo | 29°36'59.8"S 30°27'53.6"E | Symptomatic Leaves |
| 73 | CGJM 3621 | Limpopo | 29°36'59.8"S 30°27'53.6"E | Symptomatic Leaves |
| 74 | CGJM 3622 | Limpopo | 29°36'59.8"S 30°27'53.6"E | Symptomatic Leaves |
| 75 | CGJM 3623 | Limpopo | 29°36'59.8"S 30°27'53.6"E | Symptomatic Leaves |
| 76 | CGJM 2979 | Kwazulu-Natal | S 28 55'50.9'' E 030 23'20.4'' | Symptomatic Leaves |
| 77 | CGJM 3161 | Kwazulu-Natal | S 28 55'50.9'' E 030 23'20.4'' | Non-symptomatic Leaves |
| 78 | CGJM 2986 | Kwazulu-Natal | S 28 51'01.7'' E 030 06'01.5'' | Symptomatic Nuts |
| 79 | CGJM 3032 | Kwazulu-Natal | S 28 51'01.7'' E 030 06'01.5'' | Symptomatic Nuts |
| 80 | CGJM 3142 | Kwazulu-Natal | S 28 51'29.4'' E 030 05'21.3'' | Symptomatic Nuts |
| 81 | CGJM 3079 | Kwazulu-Natal | S 27 36'39.3'' E 031 28'55.2'' | Symptomatic Shoots |
| 82 | CGJM 3082 | Kwazulu-Natal | S 27 23'17.4" E 031 49'15.4" | Symptomatic Shoots |
| 83 | CGJM 3909 | Kwazulu-Natal | S 28 55'50.9'' E 030 23'20.4'' | Symptomatic Leaves |
| 84 | CGJM 3910 | Kwazulu-Natal | S 28 55'50.9'' E 030 23'20.4'' | Symptomatic Leaves |
| 85 | CGJM 3911 | Kwazulu-Natal | S 28 51'01.7'' E 030 06'01.5'' | Symptomatic Leaves |
| 86 | CGJM 3912 | Kwazulu-Natal | S 28 51'01.7'' E 030 06'01.5'' | Symptomatic Leaves |
| **Supplementary Table S1:**  (continued) |  |  |  |  |
| **Number of Samples** | **Isolates Number** | **Location** | **Coordinates** | **Pecan substrates** |
| 87 | CGJM 3913 | Kwazulu-Natal | S 27 20'49.0'' E 030 51'14.1'' | Symptomatic Leaves |
| 88 | CGJM 3914 | Kwazulu-Natal | S 27 36'39.3'' E 031 28'55.2'' | Symptomatic Leaves |
| 89 | CGJM 3923 | Kwazulu-Natal | S 27 23'17.4" E 031 49'15.4" | Symptomatic Leaves |
| 90 | CGJM 3924 | Kwazulu-Natal | S 28 55'50.9'' E 030 23'20.4'' | Symptomatic Leaves |
| 91 | CGJM 3925 | Kwazulu-Natal | S 28 55'50.9'' E 030 23'20.4'' | Symptomatic Leaves |
| 92 | CGJM 3926 | Kwazulu-Natal | S 28 51'01.7'' E 030 06'01.5'' | Symptomatic Leaves |
| 93 | CGJM 3927 | Kwazulu-Natal | S 28 51'01.7'' E 030 06'01.5'' | Symptomatic Leaves |
| 94 | CGJM 3928 | Kwazulu-Natal | S 27 20'49.0'' E 030 51'14.1'' | Symptomatic Leaves |
| 95 | CGJM 3929 | Kwazulu-Natal | S 27 36'39.3'' E 031 28'55.2'' | Symptomatic Leaves |
| 96 | CGJM 3930 | Kwazulu-Natal | S 27 23'17.4" E 031 49'15.4" | Symptomatic Leaves |
| 97 | CGJM 3934 | Kwazulu-Natal | S 28 55'50.9'' E 030 23'20.4'' | Symptomatic Leaves |
| 98 | CGJM 3935 | Kwazulu-Natal | S 28 55'50.9'' E 030 23'20.4'' | Symptomatic Leaves |
| 99 | CGJM 3936 | Kwazulu-Natal | S 28 51'01.7'' E 030 06'01.5'' | Symptomatic Nuts |
| 100 | CGJM 3938 | Kwazulu-Natal | S 28 51'01.7'' E 030 06'01.5'' | Symptomatic Nuts |
| 101 | CGJM 3939 | Kwazulu-Natal | S 27 20'49.0'' E 030 51'14.1'' | Symptomatic Leaves |
| 102 | CGJM 3940 | Kwazulu-Natal | S 27 36'39.3'' E 031 28'55.2'' | Symptomatic Leaves |
| 103 | CGJM 3941 | Kwazulu-Natal | S 27 23'17.4" E 031 49'15.4" | Symptomatic Leaves |
| 104 | CGJM 3942 | Kwazulu-Natal | S 28 55'50.9'' E 030 23'20.4'' | Symptomatic Leaves |
| 105 | CGJM 3943 | Kwazulu-Natal | S 28 55'50.9'' E 030 23'20.4'' | Symptomatic Leaves |
| 106 | CGJM 3944 | Kwazulu-Natal | S 28 51'01.7'' E 030 06'01.5'' | Symptomatic Leaves |
| 107 | CGJM 3945 | Kwazulu-Natal | S 28 51'01.7'' E 030 06'01.5'' | Symptomatic Leaves |
| 108 | CGJM 3946 | Kwazulu-Natal | S 27 20'49.0'' E 030 51'14.1'' | Symptomatic Leaves |
| 109 | CGJM 3953 | Kwazulu-Natal | S 27 36'39.3'' E 031 28'55.2'' | Symptomatic Leaves |
| 110 | CGJM 3954 | Kwazulu-Natal | S 27 23'17.4" E 031 49'15.4" | Symptomatic Leaves |
| 111 | CGJM 3959 | Kwazulu-Natal | S 28 55'50.9'' E 030 23'20.4'' | Symptomatic Leaves |
| 112 | CGJM 3960 | Kwazulu-Natal | S 28 55'50.9'' E 030 23'20.4'' | Symptomatic Leaves |
| 113 | CGJM 3089 | Eastern Cape | S 32 01'42.4'' E 025 32'52.7'' | Symptomatic Nuts |
| 114 | CGJM 3162 | Eastern Cape | S 32 01'42.4'' E 025 32'52.7'' | Symptomatic Nuts |
| 115 | CGJM 3085 | Eastern Cape | S 32 01'42.4'' E 025 32'52.7'' | Symptomatic Shoots |
| 116 | CGJM 3083 | Eastern Cape | S 32 01'42.4'' E 025 32'52.7'' | Non-symptomatic Leaves |
| 117 | CGJM 3093 | Eastern Cape | S 32 01'42.4'' E 025 32'52.7'' | Non-symptomatic Leaves |
| 118 | CGJM 3049 | Eastern Cape | S 32 01'42.4'' E 025 32'52.7'' | Symptomatic Leaves |
| 119 | CGJM 3037 | Eastern Cape | S 32 11'57.5'' E 025 38'20.9'' | Symptomatic Leaves |
| 120 | CGJM 3052 | Eastern Cape | S 32 11'57.5'' E 025 38'20.9'' | Symptomatic Leaves |
| 121 | CGJM 3048 | Eastern Cape | S 32 11'57.5'' E 025 38'20.9'' | Symptomatic Leaves |
| 122 | CGJM 3163 | Eastern Cape | S 32 11'57.5'' E 025 38'20.9'' | Symptomatic Leaves |
| 123 | CGJM 3115 | Eastern Cape | S 32 11'57.5'' E 025 38'20.9'' | Symptomatic Leaves |
| 124 | CGJM 3131 | Eastern Cape | S 32 11'57.5'' E 025 38'20.9'' | Symptomatic Leaves |
| 125 | CGJM 3164 | Eastern Cape | S 32 11'57.5'' E 025 38'20.9'' | Symptomatic Leaves |
| 126 | CGJM 3165 | Eastern Cape | S 32 11'57.5'' E 025 38'20.9'' | Symptomatic Leaves |
| 127 | CGJM 3045 | Eastern Cape | S 32 11'57.5'' E 025 38'20.9'' | Symptomatic Leaves |
| 128 | CGJM 3065 | Eastern Cape | S 32 11'57.5'' E 025 38'20.9'' | Symptomatic Leaves |
| 129 | CGJM 3039 | Eastern Cape | S 30 39'49.6'' E 026 49'36.7'' | Symptomatic Nuts |
| **Supplementary Table S1:**  (continued) |  |  |  |  |
| **Number of Samples** | **Isolates Number** | **Location** | **Coordinates** | **Pecan substrates** |
| 130 | CGJM 3030 | Eastern Cape | S 30 39'49.6'' E 026 49'36.7'' | Symptomatic Leaves |
| 131 | CGJM 3174 | Eastern Cape | S 32 11'57.5'' E 025 38'20.9'' | Non-symptomatic Leaves |
| 132 | CGJM 3158 | Eastern Cape | S 32 42'23.5'' E 026 17'16.8'' | Symptomatic Leaves |
| 133 | CGJM 3159 | Eastern Cape | S 32 44'33.9'' E 025 36'57.2'' | Non-symptomatic Leaves |
| 134 | CGJM 3175 | Eastern Cape | S 32 44'29.3'' E 025 36'15.6'' | Symptomatic Shoots |
| 135 | CGJM 3155 | Eastern Cape | S 30 39'49.6'' E 026 49'36.7'' | Symptomatic Nuts |
| 136 | CGJM 4A | Eastern Cape | S 30 39'49.6'' E 026 49'36.7'' | Symptomatic Leaves |
| 137 | CGJM 4B | Eastern Cape | S 32 11'57.5'' E 025 38'20.9'' | Non-symptomatic Leaves |
| 138 | CGJM 4C | Eastern Cape | S 32 42'23.5'' E 026 17'16.8'' | Symptomatic Leaves |
| 139 | CGJM 4D | Eastern Cape | S 32 44'33.9'' E 025 36'57.2'' | Non-symptomatic Leaves |
| 140 | CGJM 4E | Eastern Cape | S 32 44'29.3'' E 025 36'15.6'' | Symptomatic Shoots |
| 141 | CGJM 4F | Eastern Cape | S 30 39'49.6'' E 026 49'36.7'' | Symptomatic Nuts |
| 142 | CGJM 4G | Eastern Cape | S 30 39'49.6'' E 026 49'36.7'' | Symptomatic Leaves |
| 143 | CGJM 3042 | North West | S 25 47'24.5'' E 027 45'53.1'' | Non-symptomatic Leaves |
| 144 | CGJM 3166 | North West | S 25 47'22.3'' E 027 45'50.3'' | Non-symptomatic Leaves |
| 145 | CGJM 3141 | North West | S 25 13'19.1'' E 027 32'02.5'' | Non-symptomatic Leaves |
| 146 | CGJM 3054 | North West | S 25 13'19.1'' E 027 32'02.5'' | Non-symptomatic Leaves |
| 147 | CGJM 3055 | North West | S 25 13'19.1'' E 027 32'02.5'' | Non-symptomatic Leaves |
| 148 | CGJM 3073 | North West | S 25 13'19.1'' E 027 32'02.5'' | Non-symptomatic Leaves |
| 149 | CGJM 2983 | North West | S 25 13'19.1'' E 027 32'02.5'' | Non-symptomatic Leaves |
| 150 | CGJM 2956 | North West | S25 31'12.9'' E 027 47'55.7'' | Non-symptomatic Leaves |
| 151 | CGJM 3046 | North West | S 25 31'22.2'' E 027 47'45.0'' | Non-symptomatic Leaves |
| 152 | CGJM 3034 | North West | S25 31'12.9'' E 027 47'55.7'' | Non-symptomatic Leaves |
| 153 | CGJM 3139 | North West | S 25 31'22.2'' E 027 47'45.0'' | Non-symptomatic Leaves |
| 154 | CGJM 3041 | North West | S25 31'12.9'' E 027 47'55.7'' | Non-symptomatic Leaves |
| 155 | CGJM 3029 | North West | S 25 31'22.2'' E 027 47'45.0'' | Non-symptomatic Leaves |
| 156 | CGJM 3130 | North West | S 25 32'41.1'' E 027 48'57.6'' | Non-symptomatic Leaves |
| 157 | CGJM 3071 | North West | S 25 32'40.7'' E 027 48'56.5'' | Symptomatic Leaves |
| 158 | CGJM 3072 | North West | S 25 32'41.1'' E 027 48'57.6'' | Symptomatic Leaves |
| 159 | CGJM 3168 | North West | S 25 32'40.7'' E 027 48'56.5'' | Symptomatic Leaves |
| 160 | CGJM 3104 | North West | S 25 32'41.1'' E 027 48'57.6'' | Symptomatic Leaves |
| 161 | CGJM 2977 | North West | S 25 32'41.1'' E 027 48'57.6'' | Symptomatic Leaves |
| 162 | CGJM 3096 | North West | S 25 32'40.7'' E 027 48'56.5'' | Non-symptomatic Leaves |
| 163 | CGJM 3068 | North West | S 27 14'27.4" E 026 09'39.3'' | Non-symptomatic Leaves |
| 164 | CGJM 3177 | North West | S 27 14'27.4" E 026 09'39.3'' | Symptomatic Leaves |
| 165 | CGJM 3002 | North West | S 27 14'27.4" E 026 09'39.3'' | Symptomatic Leaves |
| 166 | CGJM 3003 | North West | S 27 14'27.4" E 026 09'39.3'' | Symptomatic Leaves |
| 167 | CGJM 3145 | North West | S 27 14'27.4" E 026 09'39.3'' | Symptomatic Leaves |
| 168 | CGJM 2973 | North West | S 27 14'27.4" E 026 09'39.3'' | Symptomatic Leaves |
| 169 | CGJM 2974 | North West | S 27 14'27.4" E 026 09'39.3'' | Symptomatic Leaves |
| 170 | CGJM 2975 | North West | S 27 14'27.4" E 026 09'39.3'' | Symptomatic Leaves |
| 171 | CGJM 3153 | North West | S 27 14'27.4" E 026 09'39.3'' | Symptomatic Leaves |
| 172 | CGJM 3007 | North West | S 27 14'27.4" E 026 09'39.3'' | Symptomatic Leaves |
| **Supplementary Table S1:** (continued) |  |  |  |  |
| **Number of Samples** | **Isolates Number** | **Location** | **Coordinates** | **Pecan substrates** |
| 173 | CGJM 3169 | North West | S 27 14'27.4" E 026 09'39.3'' | Symptomatic Leaves |
| 174 | CGJM 3008 | North West | S 27 14'27.4" E 026 09'39.3'' | Symptomatic Leaves |
| 175 | CGJM 3009 | North West | S 27 14'27.4" E 026 09'39.3'' | Symptomatic Leaves |
| 176 | CGJM 2960 | North West | S 27 14'27.4" E 026 09'39.3'' | Symptomatic Leaves |
| 177 | CGJM 2952 | North West | S 27 14'27.4" E 026 09'39.3'' | Non-symptomatic Leaves |
| 178 | CGJM 2961 | North West | S 27 14'27.4" E 026 09'39.3'' | Non-symptomatic Leaves |
| 179 | CGJM 2962 | North West | S 27 14'27.4" E 026 09'39.3'' | Non-symptomatic Leaves |
| 180 | CGJM 2963 | North West | S 27 14'27.4" E 026 09'39.3'' | Non-symptomatic Leaves |
| 181 | CGJM 2953 | North West | S 27 14'27.4" E 026 09'39.3'' | Non-symptomatic Leaves |
| 182 | CGJM 2970 | North West | S 27 14'27.4" E 026 09'39.3'' | Non-symptomatic Leaves |
| 183 | CGJM 3129 | North West | S 26 24'01.0" E 026 12' 32.6" | Non-symptomatic Leaves |
| 184 | CGJM 3001 | North West | S 26 24'01.0" E 026 12' 32.6" | Symptomatic Leaves |
| 185 | CGJM 2995 | North West | S 26 24'01.0" E 026 12' 32.6" | Symptomatic Leaves |
| 186 | CGJM 2992 | North West | S 26 24'01.0" E 026 12' 32.6" | Symptomatic Leaves |
| 187 | CGJM 2968 | North West | S 26 24'01.0" E 026 12' 32.6" | Symptomatic Leaves |
| 188 | CGJM 2971 | North West | S 26 24'01.0" E 026 12' 32.6" | Symptomatic Leaves |
| 189 | CGJM 2994 | North West | S 26 24'01.0" E 026 12' 32.6" | Symptomatic Leaves |
| 190 | CGJM 3011 | North West | S 26 24'01.0" E 026 12' 32.6" | Symptomatic Leaves |
| 191 | CGJM 3018 | North West | S 26 24'01.0" E 026 12' 32.6" | Symptomatic Leaves |
| 192 | CGJM 3014 | North West | S 26 24'01.0" E 026 12' 32.6" | Symptomatic Leaves |
| 193 | CGJM 3015 | North West | S 26 24'01.0" E 026 12' 32.6" | Symptomatic Leaves |
| 194 | CGJM 3013 | North West | S 26 24'01.0" E 026 12' 32.6" | Symptomatic Leaves |
| 195 | CGJM 3019 | North West | S 26 24'01.0" E 026 12' 32.6" | Symptomatic Leaves |
| 196 | CGJM 2990 | North West | S 26 24'01.0" E 026 12' 32.6" | Symptomatic Leaves |
| 197 | CGJM 2993 | North West | S 26 35'05.1" E 026 33'18.8" | Symptomatic Leaves |
| 198 | CGJM 3010 | North West | S 26 24'01.0" E 026 12' 32.6" | Symptomatic Leaves |
| 199 | CGJM 3118 | North West | S 26 24'01.0" E 026 12' 32.6" | Symptomatic Leaves |
| 200 | CGJM 2996 | North West | S 26 35'05.1" E 026 33'18.8" | Non-symptomatic Leaves |
| 201 | CGJM 2998 | North West | S 26 35'05.1" E 026 33'18.8" | Non-symptomatic Leaves |
| 202 | CGJM 2999 | North West | S 26 35'05.1" E 026 33'18.8" | Non-symptomatic Leaves |
| 203 | CGJM 3035 | North West | S 26 35'05.1" E 026 33'18.8" | Symptomatic Leaves |
| 204 | CGJM 2982 | North West | S 26 35'05.1" E 026 33'18.8" | Symptomatic Leaves |
| 205 | CGJM 3020 | North West | S 26 35'05.1" E 026 33'18.8" | Symptomatic Leaves |
| 206 | CGJM 3077 | North West | S 26 35'05.1" E 026 33'18.8" | Symptomatic Leaves |
| 207 | CGJM 3000 | North West | S 26 35'05.1" E 026 33'18.8" | Symptomatic Leaves |
| 208 | CGJM 3023 | North West | S 26 35'05.1" E 026 33'18.8" | Non-symptomatic Leaves |
| 209 | CGJM 3022 | North West | S 26 35'05.1" E 026 33'18.8" | Non-symptomatic Leaves |
| 210 | CGJM 3004 | North West | S 26 35'05.1" E 026 33'18.8" | Symptomatic Shoots |
| 211 | CGJM 3106 | North West | S 26 47'35.1'' E 026 14'37.9'' | Symptomatic Leaves |
| 212 | CGJM 3012 | North West | S 26 47'35.1'' E 026 14'37.9'' | Symptomatic Leaves |
| 213 | CGJM 3075 | North West | S 26 47'35.1'' E 026 14'37.9'' | Symptomatic Leaves |
| 214 | CGJM 3086 | North West | S 26 35'05.1" E 026 33'18.8" | Non-symptomatic Leaves |
| 215 | CGJM 3156 | North West | S 25 57'23.7" E 027 24'49.8" | Non-symptomatic Leaves |
| **Supplementary Table S1:** (continued) |  |  |  |  |
| Number of Samples | Isolates Number | Location | Coordinates | Pecan substrates |
| 216 | CGJM 3152 | North West | S 25 57'23.7" E 027 24'49.8" | Non-symptomatic Leaves |
| 217 | CGJM 3157 | North West | S 25 46'47.7'' E 027 45'43.3" | Non-symptomatic Leaves |
| 218 | CGJM 3116 | North West | S 25 46'47.7'' E 027 45'43.3" | Non-symptomatic Leaves |
| 219 | CGJM 3133 | North West | S 25 47'41.0'' E 027 45'40.7'' | Non-symptomatic Leaves |
| 220 | CGJM 3151 | North West | S 25 47'41.0'' E 027 45'40.7'' | Non-symptomatic Leaves |
| 221 | CGJM 3171 | North West | S 25 47'06.7'' E 027 44'53.6'' | Symptomatic Nuts |
| 222 | CGJM 3172 | North West | S 25 13'19.1'' E 027 32'02.5'' | Symptomatic Leaves |
| 223 | CGJM 3107 | North West | S 25 13'19.1'' E 027 32'02.5'' | Non-symptomatic Leaves |
| 224 | CGJM 3135 | North West | S 25 13'19.1'' E 027 32'02.5'' | Non-symptomatic Leaves |
| 225 | CGJM 3088 | North West | S 26 35'05.1" E 026 33'18.8" | Symptomatic Leaves |
| 226 | CGJM 3094 | North West | S 26 35'05.1" E 026 33'18.8" | Symptomatic Leaves |
| 227 | CGJM 3176 | North West | S 26 51'04.6'' E 026 13'40.1'' | Symptomatic Leaves |
| 228 | CGJM 3081 | North West | S 26 35'05.1" E 026 33'18.8" | Symptomatic Leaves |
| 229 | CGJM 3167 | North West | S 26 35'05.1" E 026 33'18.8" | Symptomatic Leaves |
| 230 | CGJM 3143 | North West | S 26 35'05.1" E 026 33'18.8" | Symptomatic Leaves |
| 231 | CGJM 5A | North West | S 26 35'05.1" E 026 33'18.8" | Non-symptomatic Leaves |
| 232 | CGJM 5B | North West | S 26 35'05.1" E 026 33'18.8" | Non-symptomatic Leaves |
| 233 | CGJM 5C | North West | S 26 35'05.1" E 026 33'18.8" | Non-symptomatic Leaves |
| 234 | CGJM 5D | North West | S 26 35'05.1" E 026 33'18.8" | Symptomatic Leaves |
| 235 | CGJM 5E | North West | S 26 35'05.1" E 026 33'18.8" | Symptomatic Leaves |
| 236 | CGJM 5F | North West | S 26 35'05.1" E 026 33'18.8" | Symptomatic Leaves |
| 237 | CGJM 5G | North West | S 26 35'05.1" E 026 33'18.8" | Symptomatic Leaves |
| 238 | CGJM 5H | North West | S 26 35'05.1" E 026 33'18.8" | Symptomatic Leaves |
| 239 | CGJM 5I | North West | S 26 35'05.1" E 026 33'18.8" | Symptomatic Leaves |
| 240 | CGJM 5J | North West | S 26 35'05.1" E 026 33'18.8" | Non-symptomatic Leaves |
| 241 | CGJM 3006 | Mpumalanga | S 25 26'14.2'' E 030 56'01.5'' | Symptomatic Leaves |
| 242 | CGJM 3038 | Mpumalanga | S 25 26'14.2'' E 030 56'01.5'' | Symptomatic Leaves |
| 243 | CGJM 2980 | Mpumalanga | S 25 26'14.2'' E 030 56'01.5'' | Symptomatic Leaves |
| 244 | CGJM 6A | Mpumalanga | S 25 26'14.2'' E 030 56'01.5'' | Symptomatic Leaves |
| 245 | CGJM 3580 | Mpumalanga | S 25 26'14.2'' E 030 56'01.5'' | Non-symptomatic Leaves |
| 246 | CGJM 3581 | Mpumalanga | S 25 26'14.2'' E 030 56'01.5'' | Non-symptomatic Leaves |
| 247 | CGJM 3582 | Mpumalanga | S 25 26'14.2'' E 030 56'01.5'' | Non-symptomatic Leaves |
| 248 | CGJM 3583 | Mpumalanga | S 25 26'14.2'' E 030 56'01.5'' | Non-symptomatic Leaves |
| 249 | CGJM 3584 | Mpumalanga | S 25 26'14.2'' E 030 56'01.5'' | Non-symptomatic Leaves |
| 250 | CGJM 3585 | Mpumalanga | S 25 26'14.2'' E 030 56'01.5'' | Non-symptomatic Leaves |
| 251 | CGJM 3586 | Mpumalanga | S 25 26'14.2'' E 030 56'01.5'' | Non-symptomatic Leaves |
| 252 | CGJM 3587 | Mpumalanga | S 25 26'14.2'' E 030 56'01.5'' | Non-symptomatic Leaves |
| 253 | CGJM 3588 | Mpumalanga | S 25 26'14.2'' E 030 56'01.5'' | Non-symptomatic Leaves |
| 254 | CGJM 3589 | Mpumalanga | S 25 26'14.2'' E 030 56'01.5'' | Non-symptomatic Leaves |
| 255 | CGJM 3590 | Mpumalanga | S 25 26'14.2'' E 030 56'01.5'' | Symptomatic Leaves |
| 256 | CGJM 3591 | Mpumalanga | S 25 26'14.2'' E 030 56'01.5'' | Symptomatic Leaves |
| 257 | CGJM 3592 | Mpumalanga | S 25 26'14.2'' E 030 56'01.5'' | Symptomatic Leaves |
| 258 | CGJM 3593 | Mpumalanga | S 25 26'14.2'' E 030 56'01.5'' | Symptomatic Leaves |
| **Supplementary Table S1:** (continued) |  |  |  |  |
| **Number of Samples** | **Isolates Number** | **Location** | **Coordinates** | **Pecan substrates** |
| 259 | CGJM 3594 | Mpumalanga | S 25 26'14.2'' E 030 56'01.5'' | Symptomatic Leaves |
| 260 | CGJM 3595 | Mpumalanga | S 25 26'14.2'' E 030 56'01.5'' | Symptomatic Leaves |
| 261 | CGJM 3596 | Mpumalanga | S 25 26'14.2'' E 030 56'01.5'' | Symptomatic Leaves |
| 262 | CGJM 3597 | Mpumalanga | S 25 26'14.2'' E 030 56'01.5'' | Symptomatic Leaves |
| 263 | CGJM 3598 | Mpumalanga | S 25 26'14.2'' E 030 56'01.5'' | Symptomatic Nuts |
| 264 | CGJM 3599 | Mpumalanga | S 25 26'14.2'' E 030 56'01.5'' | Symptomatic Nuts |
| 265 | CGJM 3600 | Mpumalanga | S 25 26'14.2'' E 030 56'01.5'' | Symptomatic Nuts |
| 266 | CGJM 3601 | Mpumalanga | S 25 26'14.2'' E 030 56'01.5'' | Symptomatic Nuts |
| 267 | CGJM 3602 | Mpumalanga | S 25 26'14.2'' E 030 56'01.5'' | Symptomatic Shoots |
| 268 | CGJM 3603 | Mpumalanga | S 25 26'14.2'' E 030 56'01.5'' | Symptomatic Shoots |
| 269 | CGJM 3604 | Mpumalanga | S 25 26'14.2'' E 030 56'01.5'' | Symptomatic Shoots |
| 270 | CGJM 3605 | Mpumalanga | S 25 26'14.2'' E 030 56'01.5'' | Symptomatic Shoots |
| 271 | CGJM 3606 | Mpumalanga | S 25 26'14.2'' E 030 56'01.5'' | Symptomatic Shoots |
| 272 | CGJM 3607 | Mpumalanga | S 25 26'14.2'' E 030 56'01.5'' | Symptomatic Shoots |
| 273 | CGJM 3608 | Mpumalanga | S 25 26'14.2'' E 030 56'01.5'' | Non-symptomatic Leaves |
| 274 | CGJM 3609 | Mpumalanga | S 25 26'14.2'' E 030 56'01.5'' | Non-symptomatic Leaves |
| 275 | CGJM 3610 | Mpumalanga | S 25 26'14.2'' E 030 56'01.5'' | Non-symptomatic Leaves |
| 276 | CGJM 3611 | Mpumalanga | S 25 26'14.2'' E 030 56'01.5'' | Non-symptomatic Leaves |
| 277 | CGJM 3612 | Mpumalanga | S 25 26'14.2'' E 030 56'01.5'' | Symptomatic Leaves |
| 278 | CGJM 3613 | Mpumalanga | S 25 26'14.2'' E 030 56'01.5'' | Symptomatic Leaves |
| 279 | CGJM 3614 | Mpumalanga | S 25 26'14.2'' E 030 56'01.5'' | Symptomatic Leaves |
| 280 | CGJM 3615 | Mpumalanga | S 25 26'14.2'' E 030 56'01.5'' | Symptomatic Leaves |
| 281 | CGJM 3652 | Mpumalanga | S 25 26'14.2'' E 030 56'01.5'' | Symptomatic Leaves |
| 282 | CGJM 3653 | Mpumalanga | S 25 26'14.2'' E 030 56'01.5'' | Symptomatic Leaves |
| 283 | CGJM 3170 | Free State | S 26 53'17.2" E 027 23'23.9" | Non-symptomatic Leaves |
| 284 | CGJM 3117 | Free State | S 26 53'17.2" E 027 23'23.9" | Non-symptomatic Leaves |
| 285 | CGJM 3146 | Free State | S 26 53'17.2" E 027 23'23.9" | Non-symptomatic Leaves |
| 286 | CGJM 3134 | Free State | S 26 51'48.9'' E 027 17'45.6'' | Symptomatic Nuts |
| 287 | CGJM 3150 | Free State | S 26 51'48.9'' E 027 17'45.6'' | Symptomatic Nuts |
| 288 | CGJM 3149 | Free State | S 26 51'48.9'' E 027 17'45.6'' | Symptomatic Nuts |
| 289 | CGJM 3173 | Free State | S 26 51'39.4'' E 027 17'52.6'' | Symptomatic Leaves |
| 290 | CGJM 3148 | Free State | S 26 51'39.4'' E 027 17'52.6'' | Symptomatic Leaves |
| 291 | CGJM 3147 | Free State | S 26 51'39.4'' E 027 17'52.6'' | Symptomatic Leaves |
| 292 | CGJM 3624 | Free State | S 28 03'21.9" E 025 04'51.0" | Non-symptomatic Leaves |
| 293 | CGJM 3625 | Free State | S 28 03'21.9" E 025 04'51.0" | Non-symptomatic Leaves |
| 294 | CGJM 3626 | Free State | S 28 03'21.9" E 025 04'51.0" | Symptomatic Nuts |
| 295 | CGJM 3627 | Free State | S 28 03'21.9" E 025 04'51.0" | Symptomatic Nuts |
| 296 | CGJM 3628 | Free State | S 28 03'21.9" E 025 04'51.0" | Symptomatic Shoots |
| 297 | CGJM 3629 | Free State | S 28 03'21.9" E 025 04'51.0" | Symptomatic Shoots |
| 298 | CGJM 3630 | Free State | S 28 03'21.9" E 025 04'51.0" | Symptomatic Leaves |
| 299 | CGJM 3631 | Free State | S 28 03'21.9" E 025 04'51.0" | Symptomatic Leaves |
| 300 | CGJM 3632 | Free State | S 28 03'21.9" E 025 04'51.0" | Non-symptomatic Leaves |
| 301 | CGJM 3633 | Free State | S 28 03'21.9" E 025 04'51.0" | Non-symptomatic Leaves |
| **Supplementary Table S1:** (continued) |  |  |  |  |
| **Number of Samples** | **Isolates Number** | **Location** | **Coordinates** | **Pecan substrates** |
| 302 | CGJM 3634 | Free State | S 28 03'21.9" E 025 04'51.0" | Symptomatic Shoots |
| 303 | CGJM 3635 | Free State | S 28 03'21.9" E 025 04'51.0" | Symptomatic Shoots |
| 304 | CGJM 3636 | Free State | S 28 03'21.9" E 025 04'51.0" | Symptomatic Leaves |
| 305 | CGJM 3637 | Free State | S 28 03'21.9" E 025 04'51.0" | Symptomatic Leaves |
| 306 | CGJM 3638 | Free State | S 28 03'21.9" E 025 04'51.0" | Non-symptomatic Leaves |
| 307 | CGJM 3639 | Free State | S 28 03'21.9" E 025 04'51.0" | Non-symptomatic Leaves |
| 308 | CGJM 3640 | Free State | S 28 03'21.9" E 025 04'51.0" | Symptomatic Leaves |
| 309 | CGJM 3641 | Free State | S 28 03'21.9" E 025 04'51.0" | Symptomatic Leaves |
| 310 | CGJM 3642 | Free State | S 28 03'21.9" E 025 04'51.0" | Symptomatic Shoots |
| 311 | CGJM 3643 | Free State | S 28 03'21.9" E 025 04'51.0" | Symptomatic Shoots |
| 312 | CGJM 3644 | Free State | S 28 03'21.9" E 025 04'51.0" | Symptomatic Nuts |
| 313 | CGJM 3645 | Free State | S 29 06'34.5'' E 024 43'23.6'' | Symptomatic Nuts |
| 314 | CGJM 3646 | Free State | S 29 06'34.5'' E 024 43'23.6'' | Symptomatic Shoots |
| 315 | CGJM 3647 | Free State | S 29 06'34.5'' E 024 43'23.6'' | Symptomatic Shoots |
| 316 | CGJM 3648 | Free State | S 29 06'34.5'' E 024 43'23.6'' | Symptomatic Leaves |
| 317 | CGJM 3649 | Free State | S 29 06'34.5'' E 024 43'23.6'' | Symptomatic Leaves |
| 318 | CGJM 3650 | Free State | S 29 06'34.5'' E 024 43'23.6'' | Symptomatic Nuts |
| 319 | CGJM 3651 | Free State | S 29 06'34.5'' E 024 43'23.6'' | Symptomatic Nuts |
| 320 | CGJM 3535 | Northern Cape | S 27 40'41.4'' E 024 46'42.6'' | Symptomatic Leaves |
| 321 | CGJM 3536 | Northern Cape | S 27 40'41.4'' E 024 46'42.6'' | Non-symptomatic Leaves |
| 322 | CGJM 3537 | Northern Cape | S 27 39'53.1" E 024 44'09.4" | Symptomatic Nuts |
| 323 | CGJM 3538 | Northern Cape | S 27 40'41.4'' E 024 46'42.6'' | Symptomatic Nuts |
| 324 | CGJM 3539 | Northern Cape | S 29 10'49.0'' E 023 43'28.3'' | Non-symptomatic Leaves |
| 325 | CGJM 3540 | Northern Cape | S 29 10'49.0'' E 023 43'28.3'' | Non-symptomatic Leaves |
| 326 | CGJM 3541 | Northern Cape | S 29 08'53.7" E 023 42'29.7" | Symptomatic Leaves |
| 327 | CGJM 3542 | Northern Cape | S 29 08'53.7" E 023 42'29.7" | Symptomatic Leaves |
| 328 | CGJM 3543 | Northern Cape | S 29 00'30.0'' E 023 52'03.9'' | Non-symptomatic Leaves |
| 329 | CGJM 3544 | Northern Cape | S 29 01'04.2" E 023 53'03.3" | Symptomatic Leaves |
| 330 | CGJM 3545 | Northern Cape | S 29 00'55.2" E 023 52'44.6" | Symptomatic Nuts |
| 331 | CGJM 3546 | Northern Cape | S 29 00'51.5" E 023 52'41.2" | Symptomatic Shoots |
| 332 | CGJM 3547 | Northern Cape | S 29 35'24.0" E 022 54'07.9" | Non-symptomatic Leaves |
| 333 | CGJM 3548 | Northern Cape | S 29 35'24.0" E 022 54'07.9" | Symptomatic Leaves |
| 334 | CGJM 3549 | Northern Cape | S 29 35'24.0" E 022 54'07.9" | Symptomatic Nuts |
| 335 | CGJM 3550 | Northern Cape | S 29 39'32.3" E 022 46'31.8" | Non-symptomatic Leaves |
| 336 | CGJM 3551 | Northern Cape | S 29 39'32.3" E 022 46'31.8" | Symptomatic Leaves |
| 337 | CGJM 3552 | Northern Cape | S 29 39'32.3" E 022 46'31.8" | Symptomatic Shoots |
| 338 | CGJM 3553 | Northern Cape | S 29 34'20.5'' E 022 51'31.2'' | Symptomatic Leaves |
| 339 | CGJM 3554 | Northern Cape | S 29 48'49.5 E 024 24'34.3" | Symptomatic Leaves |
| 340 | CGJM 3555 | Northern Cape | S 29 48'49.5 E 024 24'34.3" | Symptomatic Leaves |
| 341 | CGJM 3556 | Northern Cape | S 29 50'26.3" E 024 22'52.3" | Non-symptomatic Leaves |
| 342 | CGJM 3557 | Northern Cape | S 29 50'26.3" E 024 22'52.3" | Non-symptomatic Leaves |
| 343 | CGJM 3558 | Northern Cape | S 29 50'26.3" E 024 22'52.3" | Symptomatic Leaves |
| 344 | CGJM 3559 | Northern Cape | S 29 50'26.3" E 024 22'52.3" | Symptomatic Nuts |
| **Supplementary Table S1:** (continued) |  |  |  |  |
| **Number of Samples** | **Isolates Number** | **Location** | **Coordinates** | **Pecan substrates** |
| 345 | CGJM 3560 | Northern Cape | S 27 54'20.7'' E 024 51'31.8'' | Symptomatic Shoots |
| 346 | CGJM 3561 | Northern Cape | S 27 54'20.7'' E 024 51'31.8'' | Symptomatic Shoots |
| 347 | CGJM 3562 | Northern Cape | S 28 30'58.1" E 021 43'38.5" | Symptomatic Leaves |
| 348 | CGJM 3863 | Northern Cape | S 28 30'16.7" E 021 43'21.8" | Symptomatic Leaves |
| 349 | CGJM 3864 | Northern Cape | S 28 30'54.1" E 021 43'19.7" | Symptomatic Leaves |
| 350 | CGJM 3865 | Northern Cape | S 27 43'56.2'' E 024 46'34.0'' | Symptomatic Shoots |
| 351 | CGJM 3866 | Northern Cape | S 27 40'53.5'' E 024 43'11.6'' | Symptomatic Shoots |
| 352 | CGJM 3867 | Northern Cape | S 29 20'43.6'' E 023 07'51.6'' | Symptomatic Nuts |
| 353 | CGJM 3868 | Northern Cape | S 29 32'05.3'' E 022 59'58.0'' | Symptomatic Nuts |
| 354 | CGJM 3869 | Northern Cape | S 29 32'11.6" E 023 00'22.2" | Non-symptomatic Leaves |
| 355 | CGJM 3870 | Northern Cape | S 29 01'04.2" E 023 53'03.3" | Non-symptomatic Leaves |
| 356 | CGJM 3871 | Northern Cape | S 29 00'55.2" E 023 52'44.6" | Symptomatic Leaves |
| 357 | CGJM 3872 | Northern Cape | S 29 00'51.5" E 023 52'41.2" | Symptomatic Leaves |
| 358 | CGJM 3873 | Northern Cape | S 27 38'43.7'' E 024 45'27.2'' | Symptomatic Shoots |
| 359 | CGJM 3874 | Northern Cape | S 27 52'42.5'' E 024 47'55.5'' | Symptomatic Shoots |
| 360 | CGJM 3875 | Northern Cape | S 27 38'43.7'' E 024 45'27.2'' | Symptomatic Nuts |
| 361 | CGJM 3876 | Northern Cape | S 27 52'42.5'' E 024 47'55.5'' | Symptomatic Nuts |
| 362 | CGJM 3877 | Northern Cape | S 27 52'43.3'' E 024 47'12.3'' | Non-symptomatic Leaves |
| 363 | CGJM 3878 | Northern Cape | S 27 38'43.7'' E 024 45'27.2'' | Non-symptomatic Leaves |
| 364 | CGJM 3879 | Northern Cape | S 27 52'42.5'' E 024 47'55.5'' | Symptomatic Leaves |
|  |  |  |  |  |
