# Supplementary material for: Random amplified microsatellites (RAMS) analysis ascertains genetic variation of Alternaria alternata causing black spot disease on Carya illinoinensis in South Africa
Source: Front Genet. 2023 Sep 27;14:1213102. doi: 10.3389/fgene.2023.1213102 (PMC10569608; doi:10.3389/fgene.2023.1213102)
Supplement: Supplementary file 3 [file Image1.pdf]

Supplementary Figure

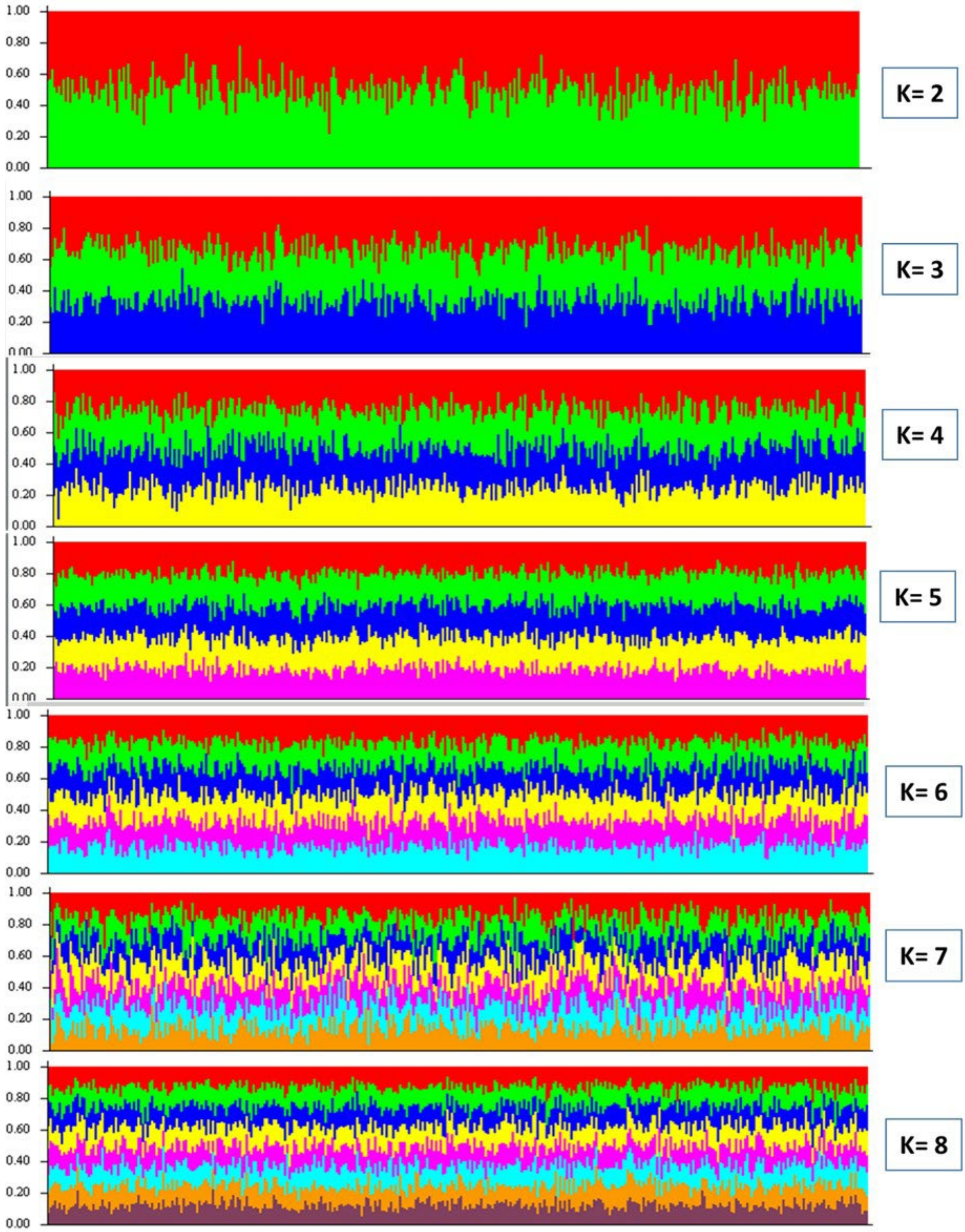

Supplementary Figure S1: Structure output for the various possible values of K = 2 - 8 for the 364 *A. alternata* isolates from eight populations Each vertical line represents one individual genotype, and each shade of colours refer to the eight populations. The proportion of each colour segment indicates the likelihood of an individual being assigned to the population represented by that colour.
