# Supplementary material for: Random amplified microsatellites (RAMS) analysis ascertains genetic variation of Alternaria alternata causing black spot disease on Carya illinoinensis in South Africa
Source: Front Genet. 2023 Sep 27;14:1213102. doi: 10.3389/fgene.2023.1213102 (PMC10569608; doi:10.3389/fgene.2023.1213102)

[illegible]

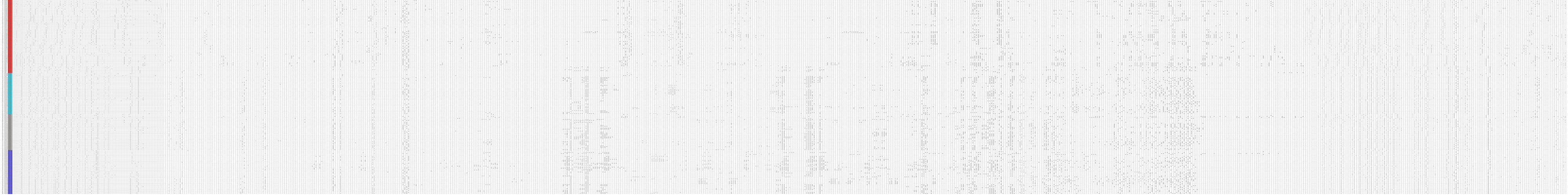

[illegible]

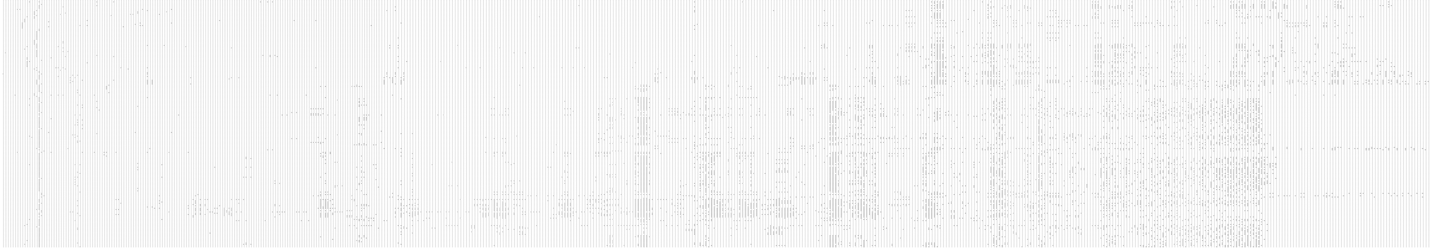

Supplement: Supplementary file 2 [file DataSheet1.PDF]
